# Short-Term Safety and Effectiveness for Tenecteplase and Alteplase in Acute Ischemic Stroke

**DOI:** 10.1001/jamanetworkopen.2025.0548

**Published:** 2025-03-12

**Authors:** Justin F. Rousseau, Jeremy M. Weber, Brooke Alhanti, Jeffrey L. Saver, Steven R. Messé, Lee H. Schwamm, Gregg C. Fonarow, Kevin N. Sheth, Eric E. Smith, Michael T. Mullen, Gisele Sampaio Silva, Brian Mac Grory, Ying Xian, Steven J. Warach

**Affiliations:** 1 Biostatistics and Clinical Informatics Section, Department of Neurology, University of Texas Southwestern Medical Center, Dallas; 2 Peter O’Donnell Jr Brain Institute, University of Texas Southwestern Medical Center, Dallas; 3 Duke Clinical Research Institute, Duke University School of Medicine, Durham, North Carolina; 4 Department of Neurology, Geffen School of Medicine at UCLA, Los Angeles, California; 5 Department of Neurology, Hospital of the University of Pennsylvania, Philadelphia; 6 Department of Neurology, Yale University School of Medicine, New Haven, Connecticut; 7 Department of Biomedical Informatics and Data Science, Yale University School of Medicine, New Haven, Connecticut; 8 Division of Cardiology, Department of Medicine, Ronald Reagan-UCLA Medical Center, Los Angeles, California; 9 Associate Section Editor, *JAMA Cardiology*; 10 Department Neurosurgery, Yale University School of Medicine, New Haven, Connecticut; 11 Department of Clinical Neurosciences, University of Calgary, Calgary, Alberta, Canada; 12 Department of Neurology, Lewis Katz School of Medicine at Temple University, Philadelphia, Pennsylvania; 13 Department of Neurology, Federal University of São Paulo (UNIFESP) and Hospital Israelita Albert Einstein, São Paulo, Brazil; 14 Duke University School of Medicine, Durham, North Carolina; 15 Department of Neurology, Dell Medical School, The University of Texas at Austin; 16 Ascension Texas, Austin, Texas

## Abstract

**Importance:**

Tenecteplase is an alternative to alteplase for emergency treatment of acute ischemic stroke. However, limited data are available comparing their clinical effectiveness in routine clinical practice.

**Objective:**

To compare short-term effectiveness and safety outcomes for patients with ischemic stroke treated with intravenous tenecteplase vs alteplase.

**Design, Setting, and Participants:**

This comparative effectiveness study used data prospectively collected from July 1, 2020, through June 30, 2022, from the Get With The Guidelines–Stroke registry.

**Exposure:**

Consecutive patients with ischemic stroke treated with either tenecteplase or alteplase within 4.5 hours from last known well time were included.

**Main Outcomes and Measures:**

The primary end point was functional independence on discharge (modified Rankin Scale [mRS] score, 0-2). Secondary effectiveness end points included disability free at discharge (mRS score, 0-1), discharge home, and independent ambulation at discharge. Safety end points included symptomatic intracranial hemorrhage (sICH) within 36 hours and combined in-hospital mortality or hospice discharge. Generalized linear mixed models were fit to evaluate associations between exposure to tenecteplase (vs alteplase) and end points after adjustment for demographic, clinical, and hospital-level variables. Adjusted odds ratios (AORs) with 95% CIs were computed.

**Results:**

Among 79 550 patients treated with intravenous thrombolysis, the mean (SD) age was 68.6 (14.8) years, 38 596 (48.5%) were female, and the median National Institutes of Health Stroke Scale (NIHSS) score was 7 (IQR, 4-13). Of these patients, 9465 (11.9%) received tenecteplase (mean [SD] age, 69.6 [14.7] years; median NIHSS score, 7 [IQR, 4-14]; 4504 [47.6%] female) and 70 085 (88.1%) received alteplase (mean [SD] age, 68.5 [14.8] years; median NIHSS score, 7 [IQR, 4-13]; 34 092 [48.6%] female). After adjustment for covariates, no significant differences were found between tenecteplase and alteplase in effectiveness or safety outcomes for the overall cohort, including functional independence at discharge (AOR, 1.00; 95% CI, 0.93-1.07), sICH (AOR, 0.96; 95% CI, 0.83-1.11), and in-hospital mortality or hospice discharge (AOR, 0.98; 95% CI, 0.89-1.07), but significant improvement was found in discharge home (AOR, 1.26; 95% CI, 1.03-1.53), in-hospital mortality (AOR, 0.63; 95% CI, 0.47-0.85), and composite in-hospital mortality or hospice discharge (AOR, 0.78; 95% CI, 0.62-0.97) among those who were eligible for but did not undergo endovascular thrombectomy.

**Conclusions and Relevance:**

This large, nationwide comparative effectiveness study using data from routine clinical practice demonstrated similar effectiveness and safety outcomes with tenecteplase compared with alteplase in patients with acute ischemic stroke. This study supports tenecteplase as a reasonable alternative to alteplase.

## Key Points

**Question:**

Is intravenous tenecteplase comparable to alteplase in effectiveness and safety for the treatment of acute ischemic stroke?

**Findings:**

This comparative effectiveness study of 79 550 patients found no significant differences between tenecteplase and alteplase on effectiveness outcomes, including functional independence at discharge, freedom from disability at discharge, discharge to home, and independent ambulation at discharge; or on safety outcomes, including symptomatic intracranial hemorrhage within 36 hours and combined in-hospital mortality or hospice discharge.

**Meaning:**

These findings suggest that tenecteplase is a reasonable alternative to alteplase in the treatment of acute ischemic stroke.

## Teaser

This comparative effectiveness study compares the clinical effectiveness and safety of tenecteplase and alteplase for the treatment of acute ischemic stroke.

## Introduction

Stroke is the third-leading cause of death and disability worldwide as of 2021 with opportunities for improvement in the quality of acute care.^1,2,3^ Tenecteplase is an alternative to alteplase for intravenous thrombolytic treatment of acute ischemic stroke. Emerging evidence from randomized clinical trials supports that tenecteplase is at least noninferior to alteplase for thrombolytic treatment of acute ischemic stroke and potentially superior for treating stroke due to large vessel occlusion (LVO).^4,5,6,7,8,9^

There is a biological basis for these clinical trial findings. Tenecteplase has greater fibrin specificity and a longer half-life than alteplase,^10^ permitting more simple preparation and administration with a single bolus for 5 to 10 seconds, whereas alteplase requires an initial bolus immediately followed by a 60-minute infusion. These differences are associated with other process-related advantages of tenecteplase over alteplase in acute ischemic stroke, in which earlier initiation of treatment is associated with better outcomes. Delays or interruptions may occur in starting alteplase infusion after bolus or during infusion of alteplase, which may lead to diminished efficacy of alteplase. In addition, alteplase may lead to delays in interfacility transfers for endovascular thrombectomy (EVT) because the 1-hour infusion associated with alteplase may need to be completed before ambulance transport when advanced life support transport is not available.^11,12,13^ There is also evidence that tenecteplase is associated with fewer dosing errors, given the relatively simple administration, compared with alteplase.^14,15^

However, clinical effectiveness and safety outcomes with tenecteplase vs alteplase are not well understood across diverse clinical settings and populations in routine clinical practice. Therefore, we sought to evaluate the clinical effectiveness and safety of tenecteplase and alteplase using data from the Get With The Guidelines (GWTG)–Stroke nationwide registry in the US. The GWTG-Stroke program enables the evaluation of clinical outcomes for tenecteplase vs alteplase over a wide range of stroke severity, prestroke disability, comorbidities, and hospital size and volume that exists in clinical practice.^16^

## Methods

For this comparative effectiveness study, we analyzed data from the nationwide GWTG-Stroke registry, which captures data on more than 60% of stroke admissions in the US.^16^ Each GWTG-Stroke participating hospital received human research approval to enroll cases without individual patient consent under the common rule or a waiver of authorization and exemption from subsequent review by their institutional review board. The Duke Clinical Research Institute serves as the data analysis center and has an agreement to analyze the aggregate deidentified data for research purposes. The institutional review board at Duke University Health approved this study. This study follows the International Society for Pharmacoeconomics and Outcomes Research (ISPOR) reporting guideline.

This comparative effectiveness study of tenecteplase and alteplase had 3 aims. The first aim was to compare effectiveness outcomes for patients with ischemic stroke treated with tenecteplase with those treated with alteplase. We hypothesized that outcomes with tenecteplase would be comparable to or better than those with alteplase with regard to the primary end point of functional independence (modified Rankin Scale [mRS] score of 0-2 at discharge). This primary end point was chosen over freedom from disability at discharge (mRS score, 0-1) as patients continue to improve after discharge.

The second aim was to compare safety outcomes for patients with ischemic stroke treated with tenecteplase with those treated with alteplase. We hypothesized that outcomes with tenecteplase would be comparable to or better than those with alteplase with regard to symptomatic intracranial hemorrhage (sICH) within 36 hours, defined by GWTG-Stroke as worsening of at least 4 points on the National Institutes of Health Stroke Scale (NIHSS) attributable to an intracranial hemorrhage; life-threatening, serious systemic hemorrhage (SSH) within 36 hours; composite of sICH or SSH; composite of inpatient mortality or discharge to hospice; and inpatient mortality.

The third aim was to compare effectiveness outcomes for patients with ischemic stroke treated with tenecteplase with those treated with alteplase for the following secondary effectiveness end points: (1) freedom from disability (mRS score, 0-1) at discharge; (2) level of disability (ordinal mRS distribution) at discharge; (3) discharge to home; (4) independent ambulation at discharge among those with independent ambulation before admission; (5) composite of discharge to home and independently ambulatory at discharge among those with independent ambulation before admission; and (6) among patients undergoing EVT, end-of-procedure achievement of successful reperfusion (modified treatment in cerebral ischemia score, 2b-3).

### Setting and Population

We analyzed consecutive patients admitted on or after July 1, 2020, and discharged by June 30, 2022, with a final discharge diagnosis of ischemic stroke. We included those patients whose stroke began before hospital arrival, with documented status of large vessels on initial imaging (anterior circulation LVO, posterior circulation LVO, or no LVO), and who were treated with alteplase or tenecteplase within 4.5 hours of last known well time. Hospitals were included if they treated 5 or more patients with alteplase or tenecteplase during the study period. Patient-level exclusion criteria were (1) discharge destination not documented, (2) left against medical advice, (3) transferred to another acute care hospital, (4) transferred in from another hospital, or (5) data on sex missing.

In addition to analyzing all patients meeting eligibility criteria, we performed prespecified subgroup analyses of all patients with an LVO who were potentially eligible for EVT and in whom EVT was performed (EVT cohort) and all patients with an LVO who were potentially eligible for EVT in whom EVT was not performed (LVO non-EVT cohort). Patients were considered potentially eligible for EVT if they presented with an LVO and an NIHSS score of 6 or higher. Reasons patients eligible for EVT may not have had EVT performed include clinical improvement after thrombolysis, significant prestroke disability, patient or family refusal, lack of availability of endovascular specialist, or advanced age.

### Statistical Analysis

To assess the association between the thrombolytic given and clinical and safety outcomes, we used mixed effects logistic regression models to compute adjusted odds ratios (AORs) with 95% CIs. A proportional odds model for ordinal mRS at discharge was planned, but it failed to converge. As a result, individual models for each mRS comparison (eg, 0 vs 1-6, 0-1 vs 2-6, and so on) were fit. Multivariable models were adjusted for patient-level and hospital-level characteristics, including demographics (age, sex, race, and ethnicity); medical history (alcohol or drug misuse, atrial fibrillation or flutter, carotid stenosis, coronary artery disease or prior myocardial infarction, COVID-19 at admission, diabetes, dyslipidemia, heart failure, hypertension, peripheral vascular disease, prior ischemic stroke, kidney insufficiency, sleep apnea, and smoker); medications used before admission (antiplatelet, anticoagulant, and antihypertensive); arrival mode (arrival via emergency medical service vs private vehicle); arrival on vs off hours (regular hours: 7 am to 6 pm, Monday-Friday, nonholiday); admission vital signs (systolic blood pressure, heart rate, and body mass index); stroke severity on presentation (NIHSS score); timing (last known well to treatment time); vessel status (anterior LVO, posterior LVO, or no LVO); potential eligibility for EVT (presence of an LVO and NIHSS score ≥6); and hospital characteristics (stroke center status, region, number of hospital beds, academic hospital, rural location, number of patients treated with thrombolytics annually, and annual volume of ischemic stroke admissions). Race and ethnicity were originally collected in the site electronic health records through various sources, including patient self-report, administrative personnel during registration process, and nursing intake forms, and were abstracted in the GWTG-Stroke data entry tool. We adjusted for race and ethnicity along with the other cofactors in our models because recent findings demonstrated that despite improvement in stroke outcomes for all races and ethnicities, disparities persist by race and ethnicity.^17^

Patient-level characteristics are summarized with mean (SD) and median (IQR) for categorical variables and number (percentage) for continuous variables, both overall and by thrombolytic received (tenecteplase vs alteplase). Standardized mean differences comparing patient characteristics and outcomes between those who received tenecteplase vs alteplase were computed, with a magnitude greater than 0.10 considered potentially important. Hospital characteristics were summarized by tenecteplase adoption status. A hospital was considered a tenecteplase adopter if 5 or more cases in the study population were treated with tenecteplase. A cutoff of 5 cases was chosen to avoid classifying a hospital as a tenecteplase adopter due to a potential data reporting error. We did not include a separate category for partial adopters due to challenges differentiating those who completely adopted tenecteplase after the beginning of the study vs those who used tenecteplase and alteplase contemporaneously.

Missing data were assumed to be missing at random. Multiple imputation using the full conditional specification method was used to impute missing values for adjustment variables with less than 25% missing.^18^ A binary indicator for whether discharge mRS score was available, EVT status (eligible for and underwent EVT, eligible for and did not undergo EVT, or not eligible for EVT), ambulatory status at discharge, sICH, and SSH were added to the imputation model. Further details of the handling of missing data are included in the eMethods in Supplement 1.

A plot of the association between time to thrombolytic initiation and probability of the combined end point of discharge to home and independent ambulation at discharge was generated. This outcome was prespecified in our statistical analysis plan to maximize the number of patients with a clinical effectiveness outcome due to missingness of the primary end point of functional independence at discharge. Estimated probabilities were generated using the adjusted model for that end point. Individual patient data were plotted as points with a LOESS (locally estimated scatterplot smoothing) regression line overlaid to visualize the trend. All analyses were performed in SAS software, version 9.4 (SAS Institute Inc) at a 2-tailed significance level of *P* < .05. Figures were made in either SAS or R, version 4.1.3 (R Foundation).

## Results 

A total of 79 550 patients (mean [SD] age, 68.6 [14.8] years; 40 954 [51.5%] male and 38 596 [48.5%] female; 2638 [3.3%] Asian, 7335 [9.2%] Hispanic, 12 621 [15.9%] non-Hispanic Black, 53 085 [66.7%] non-Hispanic White, and 3871 [4.9%] other, including American Indian or Alaska Native, Native Hawaiian or Other Pacific Islander, and unable to be determined) were treated with intravenous thrombolysis within 4.5 hours of last known well time. Selected patient demographics and clinical characteristics for all thrombolytic-treated patients stratified by thrombolytic agent are displayed in Table 1 (additional patient demographics and clinical characteristics are detailed in eTable 1 in Supplement 1). Patient demographics and clinical characteristics for the EVT performed and potentially eligible for EVT and EVT not performed cohorts stratified by thrombolytic agent are given in eTables 2 and 3 in Supplement 1. Hospital characteristics by tenecteplase adoption status are given in Table 2. A total of 352 of 1800 sites (19.6%) were categorized as an adopter of tenecteplase, including 16 sites (0.9%) that used tenecteplase instead of alteplase during the entirety of the study period. Adopters were more likely to be a comprehensive stroke center, a larger academic medical center, located in the West or Northeast, and urban. Adopters had higher annual volumes of ischemic stroke cases, patients treated with a thrombolytic, and patients treated with EVT.

**Table 1.** Characteristics of Patients Receiving Tenecteplase and Alteplase

Characteristic	No./total No. of patients (%)^a^			Standardized difference
	All eligible patients (N = 79 550)	Tenecteplase (n = 9465)	Alteplase (n = 70 085)	
Age, mean (SD), y	68.6 (14.8)	69.6 (14.7)	68.5 (14.8)	0.07
Sex				
Female	38 596/79 550 (48.5)	4504/9465 (47.6)	34 092/70 085 (48.6)	−0.02
Male	40 954/79 550 (51.5)	4961/9465 (52.4)	35 993/70 085 (51.4)	
Race and ethnicity				
Asian	2638/79 550 (3.3)	491/9465 (5.2)	2147/70 085 (3.1)	0.13
Hispanic (any race)	7335/79 550 (9.2)	902/9465 (9.5)	6433/70 085 (9.2)	
Non-Hispanic Black	12 621/79 550 (15.9)	1250/9465 (13.2)	11 371/70 085 (16.2)	
Non-Hispanic White	53 085/79 550 (66.7)	6361/9465 (67.2)	46 724/70 085 (66.7)	
Other^b^	3871/79 550 (4.9)	461/9465 (4.9)	3410/70 085 (4.9)	
Medical history				
Atrial fibrillation or flutter	10 629/79 550 (13.4)	1381/9465 (14.6)	9248/70 085 (13.2)	0.04
CAD or prior MI	15 979/79 550 (20.1)	1936/9465 (20.5)	14 043/70 085 (20.0)	0.01
Diabetes	23 811/79 550 (29.9)	2780/9465 (29.4)	21 031/70 085 (30.0)	−0.01
Dyslipidemia	39 287/79 550 (49.4)	4902/9465 (51.8)	34 385/70 085 (49.1)	0.06
Hypertension	57 914/79 550 (72.8)	6830/9465 (72.2)	51 084/70 085 (72.9)	−0.02
Prior ischemic stroke	11 026/79 550 (13.9)	1312/9465 (13.9)	9714/70 085 (13.9)	0.00
Smoker	13 553/79 550 (17.0)	1549/9465 (16.4)	12 004/70 085 (17.1)	−0.02
Medications before admission				
Antiplatelet	30 987/79 550 (39.0)	3704/9465 (39.1)	27 283/70 085 (38.9)	0.00
Anticoagulant	2626/79 550 (3.3)	334/9465 (3.5)	2292/70 085 (3.3)	0.01
Arrival information				
NIHSS score				
Mean (SD)	9.1 (7.3)	9.5 (7.6)	9.0 (7.3)	0.05
Median (IQR)	7 (4-13)	7 (4-14)	7 (4-13)	
Patient arrival				
EMS from home or scene	60 608/79 550 (76.2)	7347/9465 (77.6)	53 261/70 085 (76.0)	0.07
Private transport, taxi, other from home or scene	18 368/79 550 (23.1)	2065/9465 (21.8)	16 303/70 085 (23.3)	
Not documented or unknown	424/79 550 (0.5)	24/9465 (0.3)	400/70 085 (0.6)	
Mobile stroke unit	150/79 550 (0.2)	29/9465 (0.3)	121/70 085 (0.2)	
Time from last known well time to arrival, median (IQR), min	68.0 (44.0-113.0)	70.0 (46.0-115.0)	68.0 (44.0-112.0)	0.05
Time from last known well time to thrombolytic, median (IQR), min	124.0 (90.0-172.0)	120.0 (86.0-169.0)	124.0 (90.0-173.0)	−0.07
Stroke origin				
Large artery atherosclerosis	10 400/60 221 (17.3)	1181/7851 (15.0)	9219/52 370 (17.6)	0.10
Cardioembolism	15 390/60 221 (25.6)	2246/7851 (28.6)	13 144/52 370 (25.1)	
Small vessel occlusion	9199/60 221 (15.3)	1233/7851 (15.7)	7966/52 370 (15.2)	
Stroke of other determined origin	2370/60 221 (3.9)	346/7851 (4.4)	2024/52 370 (3.9)	
Cryptogenic stroke	22 862/60 221 (38.0)	2845/7851 (36.2)	20 017/52 370 (38.2)	
Large vessel occlusion	20 682/79 550 (26.0)	2837/9465 (30.0)	17 845/70 085 (25.5)	0.10
Potentially eligible for EVT	17 036/79 550 (21.4)	2368/9465 (25.0)	14 668/70 085 (20.9)	0.10
Potentially eligible for and EVT performed	11 315/17 036 (66.4)	1674/2368 (70.7)	9641/14 668 (65.7)	0.11
EVT performed (among all patients)	11 315/79 550 (14.2)	1674/9465 (17.7)	9641/70 085 (13.8)	0.11

Abbreviations: CAD, coronary artery disease; EMS, emergency medical services; EVT, endovascular thrombectomy; MI, myocardial infarction; NIHSS, National Institutes of Health Stroke Scale.

^a^ Unless otherwise indicated.

^b^ Includes American Indian or Alaska Native, Native Hawaiian or Other Pacific Islander, and unable to be determined.

**Table 2.** Characteristics of Tenecteplase Adopter and Nonadopter Hospitals

Characteristic	No./total No. of hospitals (%)^a^		
	Total (N = 1800)	Any tenecteplase adoption (n = 352)	No tenecteplase adoption (n = 1448)
TJC stroke center status^b^			
Neither CSC nor PSC	513/1800 (28.5)	51/352 (14.5)	462/1448 (31.9)
CSC	208/1800 (11.6)	83/352 (23.6)	125/1448 (8.6)
PSC	1079/1800 (59.9)	218/352 (61.9)	861/1448 (59.5)
No. of beds in hospital, median (IQR)	251.0 (155.0-390.0)	315.0 (198.5-487.5)	237.0 (149.0-367.0)
Academic hospitals	1070/1748 (61.2)	251/343 (73.2)	819/1405 (58.3)
Region			
Northeast	350/1800 (19.4)	79/352 (22.4)	271/1448 (18.7)
Midwest	381/1800 (21.2)	59/352 (16.8)	322/1448 (22.2)
South	689/1800 (38.3)	96/352 (27.3)	593/1448 (41.0)
West	380/1800 (21.1)	118/352 (33.5)	262/1448 (18.1)
Rural location	171/1798 (9.5)	12/352 (3.4)	159/1446 (11.0)
No. of patients treated with thrombolytics annually, median (IQR)^c^	22.7 (13.6-38.7)	34.6 (21.5-53.7)	20.5 (12.4-34.3)
Annual EVT volume, median (IQR) No.^c^	0.0 (0.0-22.9)	19.4 (0.0-58.9)	0.0 (0.0-11.0)
Annual volume of ischemic stroke admissions, median (IQR) No.	175.9 (111.3-263.0)	239.0 (155.2-357.9)	160.4 (102.4-246.5)

Abbreviations: CSC, comprehensive stroke center; EVT, endovascular thrombectomy; TJC, The Joint Commission; PSC, primary stroke center.

^a^ Unless otherwise indicated.

^b^ The CSC status is based on whether the center ever was documented in prior data harvests as CSC. The PSC status is based on whether the center was ever documented in prior data harvests as PSC and never CSC. The remaining sites were classified as neither CSC nor PSC.

^c^ Thrombolytic and EVT volume were calculated using a rolling-basis definition, and the annual volumes for each site were averaged together to calculate the summary statistics in this table.

**Figure 1.**
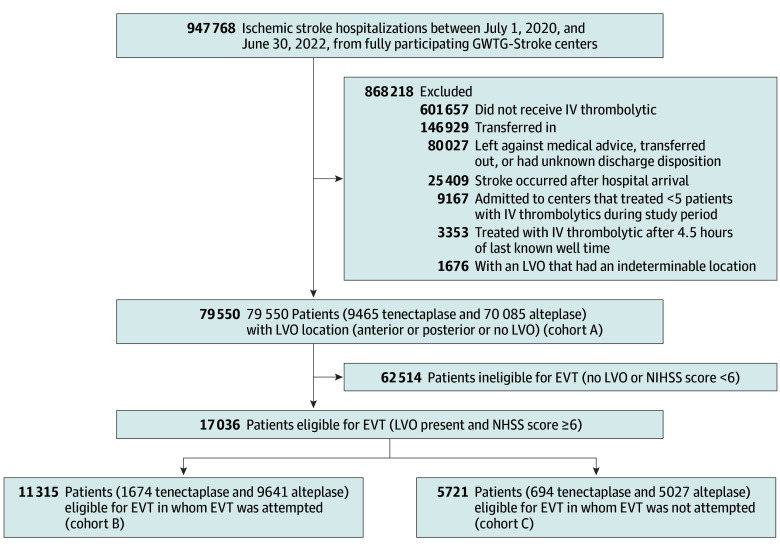
Flow Diagram of Study Population With Inclusion and Exclusion Criteria EVT indicates endovascular thrombectomy; GWTG-Stroke, Get With The Guidelines–Stroke; IV, intravenous; LVO, large vessel occlusion; NIHSS, National Institutes of Health Stroke Scale.

Among 79 550 intravenous thrombolytic-treated patients, 9465 (11.9%) received tenecteplase (mean [SD] age, 69.6 [14.7] years; 4504 [47.6%] female and 4961 [52.4%] male) and 70 085 (88.1%) received alteplase (mean [SD] age, 68.5 [14.8] years; 34 092 [48.6%] female and 35 993 [51.4%] male) (Figure 1). Among 9280 patients for whom dose per kilogram could be calculated, tenecteplase doses were between 0.20 and 0.30 mg/kg in 8934 patients (96.3%) after capping weight at 100 kg, and 127 of 9347 patients (1.4%) with dose information had documented total doses greater than 25 mg. Compared with patients receiving alteplase, patients receiving tenecteplase were more often Asian, Hispanic (any race), and non-Hispanic White and were less often non-Hispanic Black. Patients receiving tenecteplase had slightly more severe presenting deficits exhibited by higher NIHSS scores, more often had cardioembolism, and less often had a cryptogenic stroke mechanism. In addition, patients receiving tenecteplase more often had an LVO (2837 of 9465 [30.0%] vs 17 845 of 70 085 [25.5%]) and more often underwent EVT (1674 of 9465 [17.7%] vs 9641 of 70 085 [13.8%]). In contrast, age, sex, vascular risk factors, and time to treatment were similar between the tenecteplase and alteplase groups.

**Figure 2.**
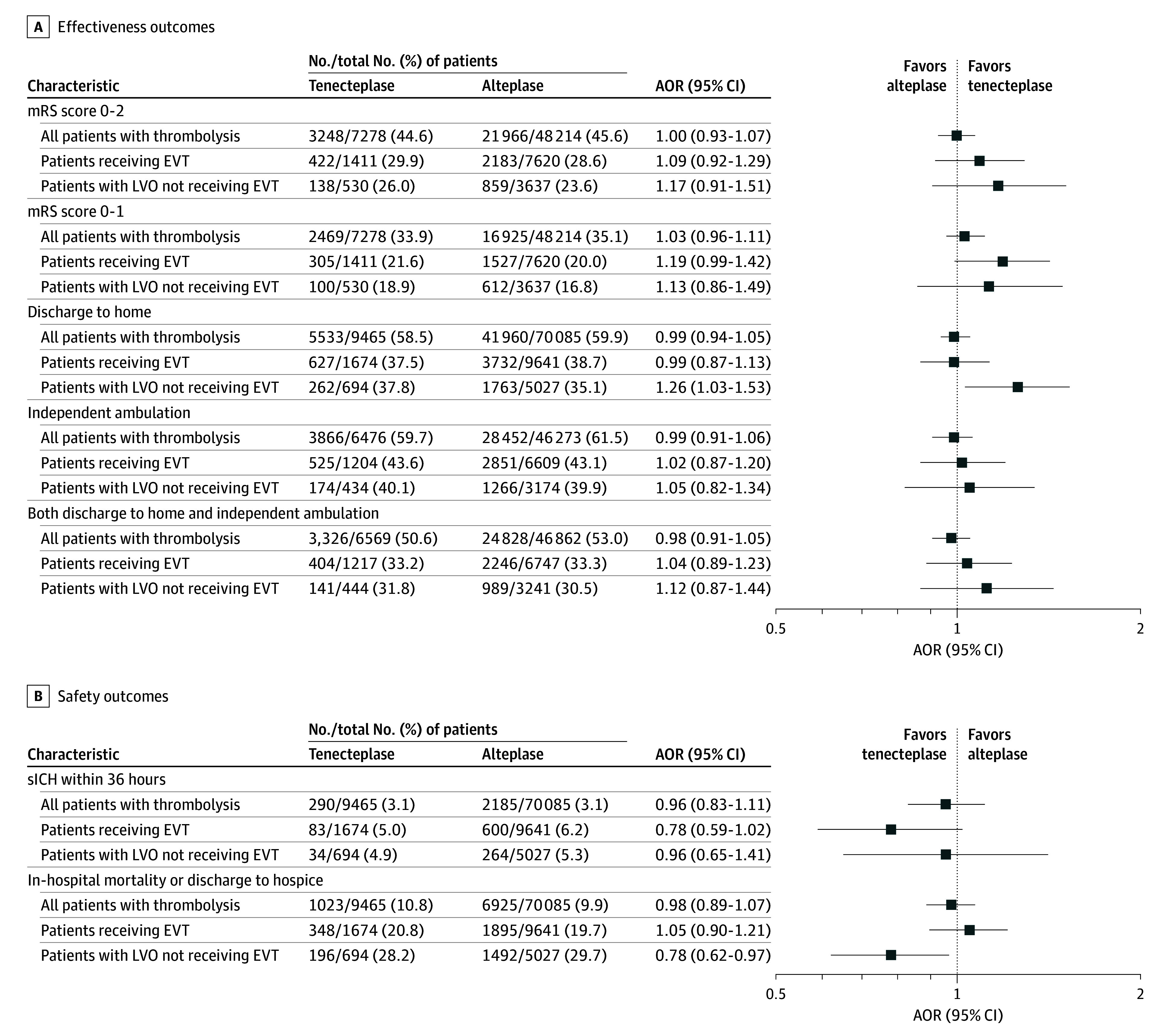
Adjusted Odds Ratios of Tenecteplase vs Alteplase AOR indicates adjusted odds ratio; EVT, endovascular thrombectomy; LVO, large vessel occlusion; sICH, symptomatic intracranial hemorrhage.

Binary effectiveness and safety outcomes among all tenecteplase-treated and alteplase-treated patients are reported in Table 3 and Figure 2. No differences were noted between tenecteplase and alteplase among all thrombolytic-treated patients in adjusted analyses for all 5 binary effectiveness end points: functional independence (mRS score, 0-2) at discharge (AOR, 1.00; 95% CI, 0.93-1.07), freedom from disability (mRS score, 0-1) at discharge (AOR, 1.03; 95% CI, 0.96-1.11), discharge to home (AOR, 0.99; 95% CI, 0.94-1.05), independent ambulation at discharge (AOR, 0.99; 95% CI, 0.91-1.06), and the combination of discharge to home and independent ambulation at discharge (AOR, 0.98; 95% CI, 0.91-1.05) (Table 3). The distribution of all 7 discharge mRS levels also did not differ (eFigure 1 in Supplement 1). Efficacy outcome information availability rates for the total population of 79 550 were as follows: discharge destination, 79 550 (100%); ambulatory status at discharge, 52 749 (66.3%) (97.0% among the 54 403 documented to ambulate independently before stroke); and discharge mRS score, 55 492 (69.8%). Patient features among those with available vs missing mRS scores are given in eTable 4 in Supplement 1. For safety outcomes, sICH (AOR, 0.96; 95% CI, 0.83-1.11), composite sICH or SSH (AOR, 0.95; 95% CI, 0.83-1.09), in-hospital mortality (AOR, 0.95; 95% CI, 0.84-1.06), and composite in-hospital mortality or discharge to hospice (AOR, 0.98; 95% CI, 0.89-1.07) rates also did not differ between the tenecteplase and alteplase groups in the overall cohort.

**Table 3.** Short-Term Effectiveness and Safety Outcomes With Tenecteplase and Alteplase Among All Thrombolytic-Treated Patients

Outcome	No./total No. of patients (%)			Odds ratio (95% CI)	
	Total	Tenecteplase	Alteplase	Unadjusted	Adjusted
Effectiveness outcomes at discharge^a^					
mRS score 0-2	25 214/55 492 (45.4)	3248/7278 (44.6)	21 966/48 214 (45.6)	0.99 (0.93-1.05)	1.00 (0.93-1.07)
mRS score 0-1	19 394/55 492 (34.9)	2469/7278 (33.9)	16 925/48 214 (35.1)	1.01 (0.94-1.07)	1.03 (0.96-1.11)
Discharge home	47 493/79 550 (59.7)	5533/9465 (58.5)	41 960/70 085 (59.9)	0.94 (0.89-0.99)	0.99 (0.94-1.05)
Independent ambulation	32 318/52 749 (61.3)	3866/6476 (59.7)	28 452/46 273 (61.5)	0.95 (0.89-1.02)	0.99 (0.91-1.06)
Both discharge home and independent ambulation	28 154/53 431 (52.7)	3326/6569 (50.6)	24 828/46 862 (53.0)	0.94 (0.88-1.00)	0.98 (0.91-1.05)
Safety outcomes					
sICH	2475/79 550 (3.1)	290/9465 (3.1)	2185/70 085 (3.1)	1.02 (0.88-1.18)	0.96 (0.83-1.11)
SSH	343/79 550 (0.4)	40/9465 (0.4)	303/70 085 (0.4)	NR^b^	NR^b^
sICH and SSH	2787/79 550 (3.5)	328/9465 (3.5)	2459/70 085 (3.5)	1.01 (0.88-1.16)	0.95 (0.83-1.09)
In-hospital mortality	3711/79 550 (4.7)	475/9465 (5.0)	3236/70 085 (4.6)	1.06 (0.94-1.20)	0.95 (0.84-1.06)
In-hospital mortality or discharge to hospice	7948/79 550 (10.0)	1023/9465 (10.8)	6925/70 085 (9.9)	1.08 (1.00-1.18)	0.98 (0.89-1.07)

Abbreviations: mRS, modified Rankin Scale; NR, not reported; sICH, symptomatic intracranial hemorrhage; SSH, serious systemic hemorrhage.

^a^ For effectiveness outcomes, data were missing in 0% of patients for discharge destination, 3.0% for ambulatory status at discharge among those documented to ambulate independently before stroke, 33.7% overall for ambulatory status at discharge, and 30.2% for mRS score at discharge. eTable 2 in Supplement 1 compares demographic and clinical characteristics of patients with nonmissing and missing mRS scores.

^b^ Models for SSH were not fit due to low event counts.

Effectiveness and safety outcomes among EVT-treated patients and EVT-eligible but not treated patients are reported in eTables 5 and 6 in Supplement 1. In adjusted analyses, discharge to home for EVT-eligible patients who did not undergo EVT was significantly improved with tenecteplase vs alteplase (AOR, 1.26; 95% CI, 1.03-1.53). None of the other effectiveness outcomes differed between the tenecteplase and alteplase cohorts in these patient subgroups. As for safety outcomes, there were significant improvements with tenecteplase in in-hospital mortality (AOR, 0.63; 95% CI, 0.47-0.85) and composite in-hospital mortality or discharge to hospice in the adjusted analysis (AOR, 0.78; 95% CI, 0.62-0.97). Among EVT-treated patients, effectiveness and safety outcomes, including successful reperfusion rates (modified treatment in cerebral ischemia score, 2b-3) (AOR, 1.03; 95% CI, 0.82-1.29), did not statistically differ between the 2 thrombolytic groups.

eFigure 2 in Supplement 1 shows the adjusted association between the last known well time to thrombolytic initiation and the probability of the combination of discharge to home and ambulating independently at discharge. For both the tenecteplase and alteplase cohorts, the probability of the combination of discharge to home and ambulating independently at discharge decreased with increasing time from last known well time to agent administration.

## Discussion

In this comparative effectiveness study of patients treated with intravenous thrombolysis in broad clinical practice, comparable short-term clinical effectiveness outcomes were found in tenecteplase vs alteplase at discharge, including freedom from disability, functional independence, independent ambulation, and discharge to home. Comparable short-term safety outcomes were demonstrated, including sICH and combined in-hospital mortality or discharge to hospice. Discharge to home was the only effectiveness outcome that showed statistically significant improvement in patients who were EVT eligible but did not undergo EVT. In this subgroup as well as in patients who underwent EVT, no other clinical effectiveness differences were seen with tenecteplase vs alteplase, including no differences in successful reperfusion rates among patients undergoing EVT. However, there were statistically significant improved safety outcomes, including in-hospital mortality and composite in-hospital mortality or discharge to hospice, among those who were potentially eligible but did not have EVT performed.

These findings are consonant with and importantly extend prior findings demonstrating that tenecteplase is noninferior to alteplase for emergency intravenous treatment of acute ischemic stroke. In a meta-analysis of 10 randomized clinical trials comparing tenecteplase and alteplase among 5123 patients (2677 receiving tenecteplase and 2446 receiving alteplase) with acute ischemic stroke, no significant difference in functional outcomes at 90 days was observed.^8^ However, tenecteplase was associated with improved recanalization rates and significant improvement in excellent functional outcome at 90 days when an LVO was present. Furthermore, including findings from another meta-analysis, there were no significant differences in rates of sICH or mortality between the 2 thrombolytics.^8,19^ The European Stroke Organization, supported by a meta-analysis of 7 randomized clinical trials from the above meta-analyses, recommended tenecteplase as a safe and effective alternative to alteplase and provided an expert consensus statement recommending tenecteplase over alteplase for patients with acute ischemic stroke within 4.5 hours of symptom onset.^20^ Our study also found similar effectiveness and safety outcomes for tenecteplase and alteplase in broad, routine clinical practice. The findings from our study contrast with pooled evidence from randomized clinical trials suggesting that tenecteplase may be superior to alteplase^21^ along with some prior large observational studies comparing the effectiveness and safety of tenecteplase vs alteplase for treatment of acute ischemic stroke that found some signals of superior outcomes with tenecteplase over alteplase but also had design constraints.^22^ The CERTAIN (Comparative Effectiveness of Routine Tenecteplase vs Alteplase in Acute Ischemic Stroke) collaborative registry evaluated contemporaneous patients and found in adjusted analyses reduced odds of sICH with tenecteplase but no difference in mortality.^23^ That study did not assess functional outcomes.

### Limitations

This study has limitations. First, it is an observational study without randomizations, and registry data are self-reported with outcomes evaluated from personnel not blinded to the treatment. Despite adjustment for multiple patient characteristics, including stroke severity, and for hospital characteristics, there is the potential for residual measured and unmeasured confounding as well as potential for spurious results due to multiple models and covariates. One potential source of variance not adjusted for was the variability in tenecteplase dosing across sites. Second, due to limited data collected from transferring hospitals and an inability to link patient data across hospitals, we did not include interfacility transfer patients, precluding detection of clinical outcomes arising from potentially faster transfers with bolus dose tenecteplase than hourlong infusion alteplase. Third, this analysis included only patients who had final discharge diagnoses of acute ischemic stroke. It is possible that the analysis missed some patients coded as having a transient ischemic attack because their deficits cleared after thrombolytic treatment within 24 hours of onset. However, Centers for Medicare & Medicaid instructions to hospitals are to code such patients as having ischemic stroke (averted) rather than transient ischemic attack, mitigating the risk of substantial miscoding. Fourth, due to high rates of missingness, we were not able to include 90-day outcomes in our evaluation of clinical effectiveness, but discharge outcomes have been shown to correlate well with 90-day outcomes.^24,25^ Fifth, GWTG-Stroke is a voluntary program in the US, limiting generalizability to nonparticipating hospitals or health systems in other countries. Sixth, evaluation for LVO via vascular imaging is not routinely assessed in all centers for all patients being evaluated for stroke, which may lead to selection bias. Seventh, despite inclusion of 352 of 1800 sites (19.6%) having at least partially adopted tenecteplase, there may be unmeasured confounding associated with early adopter sites.

## Conclusions

This large, nationwide comparative effectiveness study using data from routine clinical practice demonstrated similar short-term safety and effectiveness outcomes with tenecteplase compared to alteplase in patients with acute ischemic stroke. This study supports tenecteplase as a reasonable alternative to alteplase, with practical advantages for tenecteplase preparation and administration. As the adoption of tenecteplase increases across stroke center types, geographic regions, and stroke characteristics, future research will provide additional insight into whether small but clinically important differences in outcomes between tenecteplase and alteplase exist.

## Notes

Supplement 1. **eMethods.** Supplemental Methods **eFigure 1.** Stacked Bar Chart of mRS at Discharge by IV Thrombolytic Received **eFigure 2.** Relationship of Time to IV Thrombolytic Initiation From Last Known Well Time to Probability of a Composite of Discharge to Home and Independent Ambulation at Discharge **eTable 1.** All Patient Demographics and Characteristics for All Eligible Patients **eTable 2.** Demographic Table for EVT Cohort (Potentially Eligible for and EVT Performed) Is Presented Below **eTable 3.** Demographic Table for LVO Non-EVT Cohort (Potentially Eligible for EVT and EVT Not Performed) Is Presented Below **eTable 4.** Comparison of Patient Characteristics Between Those With Discharge mRS Available vs. Missing **eTable 5.** Effectiveness and Safety Outcomes with Tenecteplase and Alteplase Among Potentially EVT-Eligible Patients Treated With EVT (EVT Cohort) **eTable 6.** Effectiveness and Safety Outcomes With Tenecteplase and Alteplase Among Potentially EVT-Eligible Patients Not Treated With EVT (LVO Non-EVT Cohort)

Supplement 2.
Data Sharing Statement

## References

[1] Naghavi M, Ong KL, Aali A, ; GBD 2021 Causes of Death Collaborators. Global burden of 288 causes of death and life expectancy decomposition in 204 countries and territories and 811 subnational locations, 1990-2021: a systematic analysis for the Global Burden of Disease Study 2021. Lancet. 2024;403(10440):2100-2132. doi:10.1016/S0140-6736(24)00367-2 38582094 PMC11126520

[2] Ferrari AJ, Santomauro DF, Aali A, ; GBD 2021 Diseases and Injuries Collaborators. Global incidence, prevalence, years lived with disability (YLDs), disability-adjusted life-years (DALYs), and healthy life expectancy (HALE) for 371 diseases and injuries in 204 countries and territories and 811 subnational locations, 1990-2021: a systematic analysis for the Global Burden of Disease Study 2021. Lancet. 2024;403(10440):2133-2161. doi:10.1016/S0140-6736(24)00757-8 38642570 PMC11122111

[3] Latorre JGS, Flanagan S, Phipps MS, Shenoy AM, Bennett A, Seidenwurm D. Quality improvement in neurology. Neurology. 2017;89(15):1619-1626. doi:10.1212/WNL.0000000000004486 28904088

[4] Burgos AM, Saver JL. Evidence that tenecteplase is noninferior to alteplase for acute ischemic stroke: meta-analysis of 5 randomized trials. Stroke. 2019;50(8):2156-2162. doi:10.1161/STROKEAHA.119.025080 31318627

[5] Katsanos AH, Safouris A, Sarraj A, . Intravenous thrombolysis with tenecteplase in patients with large vessel occlusions: systematic review and meta-analysis. Stroke. 2021;52(1):308-312. doi:10.1161/STROKEAHA.120.030220 33272127

[6] Warach SJ, Dula AN, Milling TJ Jr. Tenecteplase thrombolysis for acute ischemic stroke. Stroke. 2020;51(11):3440-3451. doi:10.1161/STROKEAHA.120.029749 33045929 PMC7606819

[7] Wang Y, Li S, Pan Y, ; TRACE-2 Investigators. Tenecteplase versus alteplase in acute ischaemic cerebrovascular events (TRACE-2): a phase 3, multicentre, open-label, randomised controlled, non-inferiority trial. Lancet. 2023;401(10377):645-654. doi:10.1016/S0140-6736(22)02600-9 36774935

[8] Huang J, Zheng H, Zhu X, Zhang K, Ping X. Tenecteplase versus alteplase for the treatment of acute ischemic stroke: a meta-analysis of randomized controlled trials. Ann Med. 2024;56(1):2320285. doi:10.1080/07853890.2024.2320285 38442293 PMC10916912

[9] Parsons MW, Yogendrakumar V, Churilov L, ; TASTE Investigators. Tenecteplase versus alteplase for thrombolysis in patients selected by use of perfusion imaging within 4·5 h of onset of ischaemic stroke (TASTE): a multicentre, randomised, controlled, phase 3 non-inferiority trial. Lancet Neurol. 2024;23(8):775-786. doi:10.1016/S1474-4422(24)00206-0 38880118

[10] Davydov L, Cheng JW. Tenecteplase: a review. Clin Ther. 2001;23(7):982-997. doi:10.1016/S0149-2918(01)80086-2 11519775

[11] Zachrison KS, Schwamm LH. The promise of tenecteplase in acute stroke: within reach or beyond approval? Med. 2022;3(10):651-655. doi:10.1016/j.medj.2022.09.005 36202099

[12] Acheampong P, May MT, Ford GA, Dixit AK. Bolus-infusion delays of alteplase during thrombolysis in acute ischaemic stroke and functional outcome at 3 months. Stroke Res Treat. 2014;2014:358640. doi:10.1155/2014/358640 24876988 PMC4021679

[13] Brodoehl S, Günther A, Witte OW, Klingner CM. How to manage thrombolysis interruptions in acute stroke? Clin Neuropharmacol. 2015;38(3):85-88. doi:10.1097/WNF.0000000000000081 25970276

[14] Mehta RH, Alexander JH, Van de Werf F, . Relationship of incorrect dosing of fibrinolytic therapy and clinical outcomes. JAMA. 2005;293(14):1746-1750. doi:10.1001/jama.293.14.1746 15827313

[15] Murphy SA, Gibson CM, Van de Werf F, McCabe CH, Cannon CP. Comparison of errors in estimating weight and in dosing of single-bolus tenecteplase with tissue plasminogen activator (TIMI 10B and ASSENT I). Am J Cardiol. 2002;90(1):51-54. doi:10.1016/S0002-9149(02)02387-1 12088781

[16] Ormseth CH, Sheth KN, Saver JL, Fonarow GC, Schwamm LH. The American Heart Association’s Get With the Guidelines (GWTG)–Stroke development and impact on stroke care. Stroke Vasc Neurol. 2017;2(2):94-105. doi:10.1136/svn-2017-000092 28959497 PMC5600018

[17] Man S, Solomon N, Mac Grory B, . Trends in stroke thrombolysis care metrics and outcomes by race and ethnicity, 2003-2021. JAMA Netw Open. 2024;7(2):e2352927. doi:10.1001/jamanetworkopen.2023.52927 38324315 PMC10851100

[18] van Buuren S. Multiple imputation of discrete and continuous data by fully conditional specification. Stat Methods Med Res. 2007;16(3):219-242. doi:10.1177/0962280206074463 17621469

[19] Wang Y, Cai X, Fang Q, Zhu J. Efficacy and safety outcomes of tenecteplase versus alteplase for thrombolysis of acute ischemic stroke: a meta-analysis of 9 randomized controlled trials. J Neurol Sci. 2024;458:122912. doi:10.1016/j.jns.2024.122912 38325064

[20] Alamowitch S, Turc G, Palaiodimou L, . European Stroke Organisation (ESO) expedited recommendation on tenecteplase for acute ischaemic stroke. Eur Stroke J. 2023;8(1):8-54. doi:10.1177/23969873221150022 37021186 PMC10069183

[21] Wang L, Hao M, Wu N, Wu S, Fisher M, Xiong Y. Comprehensive review of tenecteplase for thrombolysis in acute ischemic stroke. J Am Heart Assoc. 2024;13(9):e031692. doi:10.1161/JAHA.123.031692 38686848 PMC11179942

[22] Katsanos AH, Psychogios K, Turc G, . Off-label use of tenecteplase for the treatment of acute ischemic stroke: a systematic review and meta-analysis. JAMA Netw Open. 2022;5(3):e224506. doi:10.1001/jamanetworkopen.2022.4506 35357458 PMC8972028

[23] Warach SJ, Ranta A, Kim J, . Symptomatic intracranial hemorrhage with tenecteplase vs alteplase in patients with acute ischemic stroke: the Comparative Effectiveness of Routine Tenecteplase vs Alteplase in Acute Ischemic Stroke (CERTAIN) Collaboration. JAMA Neurol. 2023;80(7):732-738. doi:10.1001/jamaneurol.2023.1449 37252708 PMC10230371

[24] Zhang Q, Yang Y, Saver JL. Discharge destination after acute hospitalization strongly predicts three month disability outcome in ischemic stroke. Restor Neurol Neurosci. 2015;33(5):771-775. doi:10.3233/RNN-150531 26410209

[25] Ovbiagele B, Saver JL. Day-90 acute ischemic stroke outcomes can be derived from early functional activity level. Cerebrovasc Dis. 2010;29(1):50-56. doi:10.1159/000255974 19893312

