# Error in Figure 2

**DOI:** 10.1001/jamanetworkopen.2025.24999

**Published:** 2025-07-08

In the Original Investigation titled “Short-Term Safety and Effectiveness for Tenecteplase and Alteplase in Acute Ischemic Stroke,”^1^ published March 12, 2025, in Figure 2, the “Favors alteplase” and “Favors tenecteplase” labels above the graph were incorrectly applied to the full list of outcomes. The graph has been revised to correctly apply the labels to the effectiveness outcomes and safety outcomes. This article has been corrected.^1^
