# Supplementary material for: Short-Term Safety and Effectiveness for Tenecteplase and Alteplase in Acute Ischemic Stroke
Source: JAMA Netw Open. 2025 Mar 12;8(3):e250548. doi: 10.1001/jamanetworkopen.2025.0548 (PMC11904722; doi:10.1001/jamanetworkopen.2025.0548)
Supplement: Supplement 1. — eMethods. Supplemental Methods eFigure 1. Stacked Bar Chart of mRS at Discharge by IV Thrombolytic Received eFigure 2. Relationship of Time to IV Thrombolytic Initiation From Last Known Well Time to Probability of a Composite of Discharge to Home and Independent Ambulation at Discharge eTable 1. All Patient Demographics and Characteristics for All Eligible Patients eTable 2. Demographic Table for EVT Cohort (Potentially Eligible for and EVT Performed) Is Presented Below eTable 3. Demographic Table for LVO Non-EVT Cohort (Potentially Eligible for EVT and EVT Not Performed) Is Presented Below eTable 4. Comparison of Patient Characteristics Between Those With Discharge mRS Available vs. Missing eTable 5. Effectiveness and Safety Outcomes with Tenecteplase and Alteplase Among Potentially EVT-Eligible Patients Treated With EVT (EVT Cohort) eTable 6. Effectiveness and Safety Outcomes With Tenecteplase and Alteplase Among Potentially EVT-Eligible Patients Not Treated With EVT (LVO Non-EVT Cohort) [file jamanetwopen-e250548-s001.pdf]

Supplemental Online Content

Rousseau JF, Weber JM, Alhanti B, et al. Short-term safety and effectiveness for Tenecteplase and alteplase in acute ischemic stroke. *JAMA Netw Open*. Published online March 12, 2025. doi:10.1001/jamanetworkopen.2025.0548

eMethods. Supplemental Methods
eFigure 1. Stacked Bar Chart of mRS at Discharge by IV Thrombolytic Received
eFigure 2. Relationship of Time to IV Thrombolytic Initiation From Last Known Well to Probability of a Composite of Discharge to Home and Independent Ambulation at Discharge
eTable 1. All Patient Demographics and Characteristics for All Eligible Patients
eTable 2. Demographic Table for EVT Cohort (Potentially Eligible for and EVT Performed) Is Presented Below
eTable 3. Demographic Table for LVO Non-EVT Cohort (Potentially Eligible for EVT and EVT Not Performed) Is Presented Below
eTable 4. Comparison of Patient Characteristics Between Those With Discharge mRS Available vs. Missing
eTable 5. Effectiveness and Safety Outcomes with Tenecteplase and Alteplase Among Potentially EVT-Eligible Patients Treated With EVT (EVT Cohort)
eTable 6. Effectiveness and Safety Outcomes With Tenecteplase and Alteplase Among Potentially EVT-Eligible Patients Not Treated With EVT (LVO Non-EVT Cohort)

This supplemental material has been provided by the authors to give readers additional information about their work.

© 2025 Rousseau JF et al. *JAMA Network Open*.

eMethods. Supplemental Methods

Handling of Missing Data:

We made the following assumptions to handle missing data. Missingness in medical history variables were imputed to “No”. Missing hospital location (rural vs urban) were imputed to urban. Missing academic hospital were imputed to “non-academic hospital”. Missing number of beds were imputed to 210 for non-academic hospitals and 430 for academic hospitals. Otherwise, hospital characteristics were not imputed. Patients with an in-hospital death had their mRS at discharge set to 6 if not already done. Patients without vascular imaging performed were assumed to not have an LVO. Patients with an unknown LVO location (anterior or posterior) were excluded. Missingness in thrombolytic complications (symptomatic intracranial hemorrhage and life-threatening, serious systemic hemorrhage) were assumed to be non-events.

We used 25% proportion of missingness as a guideline for whether a variable should be imputed or excluded from the analysis and discussed cases close to the threshold. For variables determined to be imputed based on our 25% missingness guideline, multiple imputation was used. Missing data in variables that are only described (i.e. not used in models) were not imputed. Six variables included in adjusted models had missing values (BMI 14.5%, antihypertensive usage 13.8%, NIHSS 0.2%, arrival by EMS 0.5%, systolic blood pressure 4.2%, heart rate 25.1%). Although heart rate was missing in 25.1% of cases in the overall cohort, it was missing in less than 25% in the two EVT eligible cohorts (23.8% and 20.5% missingness), and thus we decided to include the variable in multiple imputation. The distribution of these six variables was very similar between the original pre-imputed dataset and several randomly selected datasets created by multiple imputation. Standardized differences post-imputation for these six variables were also nearly identical to the original pre-imputed data. Baseline characteristics were tabulated comparing those with and without available discharge mRS (Table S2) to determine if there are differences between those two groups.

© 2025 Rousseau JF et al. *JAMA Network Open*.

eFigure 1. Stacked bar chart of mRS at discharge by IV thrombolytic received

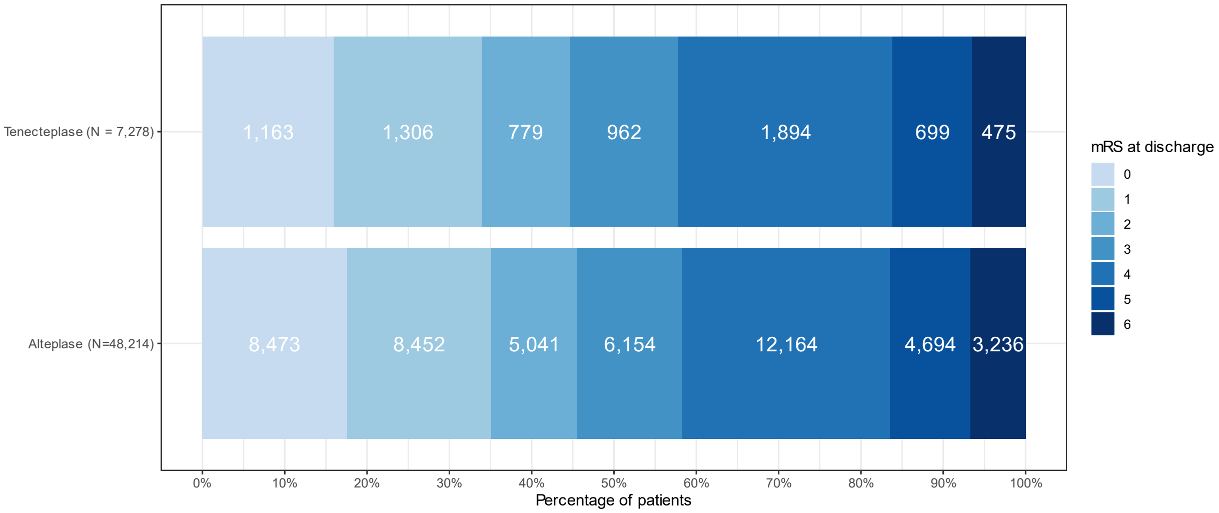

© 2025 Rousseau JF et al. *JAMA Network Open*.

eFigure 2. Relationship of time to IV thrombolytic initiation from last known well to probability of a composite of discharge to home and independent ambulation at discharge

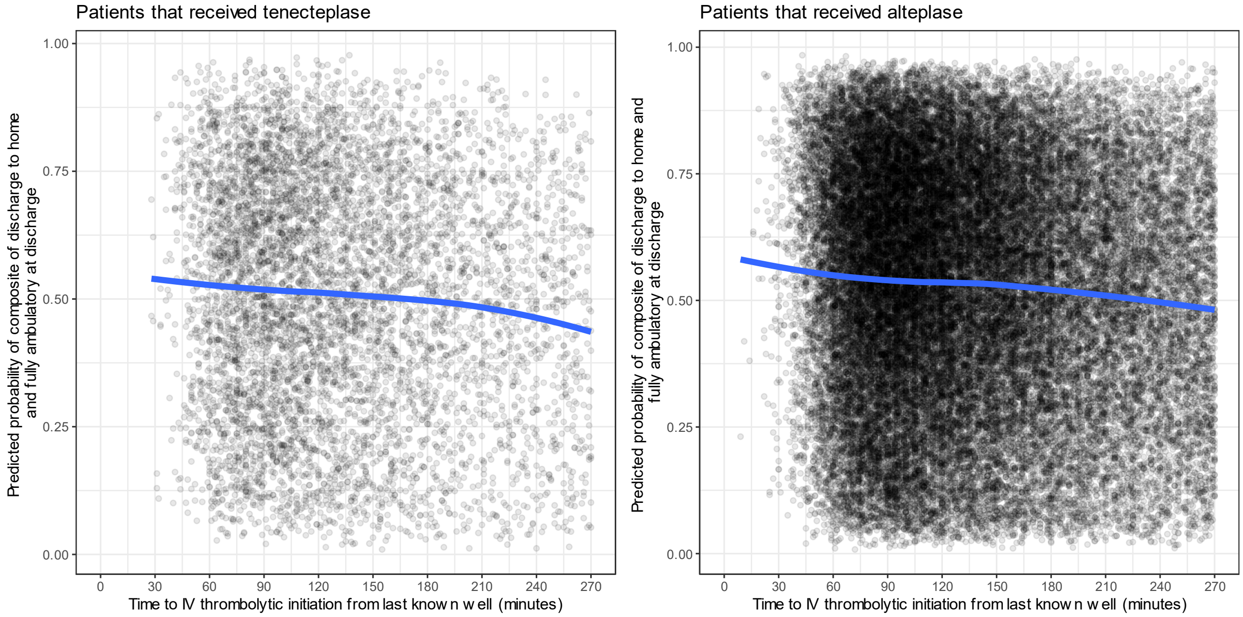

© 2025 Rousseau JF et al. *JAMA Network Open*.

eTable 1. All patient demographics and characteristics for all eligible patients

	**All patients (N=79550)**	**Tenecteplase (N=9465)**	**Alteplase (N=70085)**	**Standardized difference**
**Age (years)**				0.07
N (missing)	79550 (0)	9465 (0)	70085 (0)	
Mean (SD)	68.6 (14.8)	69.6 (14.7)	68.5 (14.8)	
Median (25th, 75th)	70.0 (59.0, 80.0)	71.0 (60.0, 81.0)	70.0 (59.0, 80.0)	
**Sex**				-0.02
Female	38596/79550 (48.5%)	4504/9465 (47.6%)	34092/70085 (48.6%)	
Male	40954/79550 (51.5%)	4961/9465 (52.4%)	35993/70085 (51.4%)	
**Race/ethnicity**				0.13
White	53085/79550 (66.7%)	6361/9465 (67.2%)	46724/70085 (66.7%)	
Black	12621/79550 (15.9%)	1250/9465 (13.2%)	11371/70085 (16.2%)	
Hispanic (any race)	7335/79550 (9.2%)	902/9465 (9.5%)	6433/70085 (9.2%)	
Asian	2638/79550 (3.3%)	491/9465 (5.2%)	2147/70085 (3.1%)	
Other (includes unable to be determined)	3871/79550 (4.9%)	461/9465 (4.9%)	3410/70085 (4.9%)	
**Body mass index (kg/m^2)**				-0.018
N (missing)	67991 (11559)	8218 (1247)	59773 (10312)	
Mean (SD)	29.3 (7.1)	29.1 (7.0)	29.3 (7.1)	
Median (25th, 75th)	28.3 (24.5, 32.9)	28.2 (24.5, 32.7)	28.3 (24.5, 32.9)	
Range	(10.0, 97.8)	(10.7, 86.3)	(10.0, 97.8)	
**Homeless**	77/79550 (0.1%)	10/9465 (0.1%)	67/70085 (0.1%)	0.00
**Insurance type**				0.09
Private/VA/Champus/Other insurance	25123/79550 (31.6%)	2814/9465 (29.7%)	22309/70085 (31.8%)	
Medicaid	9499/79550 (11.9%)	1150/9465 (12.2%)	8349/70085 (11.9%)	
Medicare	28669/79550 (36.0%)	3728/9465 (39.4%)	24941/70085 (35.6%)	
Self-pay/No insurance	3220/79550 (4.0%)	303/9465 (3.2%)	2917/70085 (4.2%)	
Other/Not documented/Unable to determine	13039/79550 (16.4%)	1470/9465 (15.5%)	11569/70085 (16.5%)	
**Past medical history**				

© 2025 Rousseau JF et al. *JAMA Network Open*.

	All patients (N=79550)	Tenecteplase (N=9465)	Alteplase (N=70085)	Standardized difference
Ambulatory status before current event				0.081
Able to ambulate independently (no help from another person) w/ or w/o device	54403/79550 (68.4%)	6728/9465 (71.1%)	47675/70085 (68.0%)	
With assistance (from person)	2918/79550 (3.7%)	384/9465 (4.1%)	2534/70085 (3.6%)	
Unable to ambulate	1104/79550 (1.4%)	126/9465 (1.3%)	978/70085 (1.4%)	
Not documented	21125/79550 (26.6%)	2227/9465 (23.5%)	18898/70085 (27.0%)	
Alcohol/drug use disorder	6285/79550 (7.9%)	750/9465 (7.9%)	5535/70085 (7.9%)	0.00
Atrial fib/flutter	10629/79550 (13.4%)	1381/9465 (14.6%)	9248/70085 (13.2%)	0.04
Carotid stenosis	2212/79550 (2.8%)	318/9465 (3.4%)	1894/70085 (2.7%)	0.04
CAD/prior MI	15979/79550 (20.1%)	1936/9465 (20.5%)	14043/70085 (20.0%)	0.01
Diabetes mellitus	23811/79550 (29.9%)	2780/9465 (29.4%)	21031/70085 (30.0%)	-0.01
Dyslipidemia	39287/79550 (49.4%)	4902/9465 (51.8%)	34385/70085 (49.1%)	0.06
Heart failure	7171/79550 (9.0%)	892/9465 (9.4%)	6279/70085 (9.0%)	0.02
Hypertension	57914/79550 (72.8%)	6830/9465 (72.2%)	51084/70085 (72.9%)	-0.02
Peripheral vascular disease	2396/79550 (3.0%)	308/9465 (3.3%)	2088/70085 (3.0%)	0.02
Prior ischemic stroke	11026/79550 (13.9%)	1312/9465 (13.9%)	9714/70085 (13.9%)	0.00
Renal insufficiency	7012/79550 (8.8%)	919/9465 (9.7%)	6093/70085 (8.7%)	0.04
Sleep apnea	5458/79550 (6.9%)	761/9465 (8.0%)	4697/70085 (6.7%)	0.05
Smoker	13553/79550 (17.0%)	1549/9465 (16.4%)	12004/70085 (17.1%)	-0.02
**Medications before admission**				
Antiplatelet	30987/79550 (39.0%)	3704/9465 (39.1%)	27283/70085 (38.9%)	0.00
Anticoagulant	2626/79550 (3.3%)	334/9465 (3.5%)	2292/70085 (3.3%)	0.01
Antihypertensive	41373/68598 (60.3%)	4798/7995 (60.0%)	36575/60603 (60.4%)	-0.01
**Arrival information**				
NIHSS				0.050
N (missing)	79374 (176)	9449 (16)	69925 (160)	

© 2025 Rousseau JF et al. *JAMA Network Open*.

	All patients (N=79550)	Tenecteplase (N=9465)	Alteplase (N=70085)	Standardized difference
Mean (SD)	9.1 (7.3)	9.5 (7.6)	9.0 (7.3)	
Median (25th, 75th)	7.0 (4.0, 13.0)	7.0 (4.0, 14.0)	7.0 (4.0, 13.0)	
Off-hour arrival (Regular Hour: 7AM-6PM, M-F, non-holiday)	42876/79550 (53.9%)	5178/9465 (54.7%)	37698/70085 (53.8%)	0.02
Patient arrival				0.07
EMS from home/scene	60608/79550 (76.2%)	7347/9465 (77.6%)	53261/70085 (76.0%)	
Private transport/taxi/other from home/scene	18368/79550 (23.1%)	2065/9465 (21.8%)	16303/70085 (23.3%)	
Not documented or unknown	424/79550 (0.5%)	24/9465 (0.3%)	400/70085 (0.6%)	
Mobile Stroke Unit	150/79550 (0.2%)	29/9465 (0.3%)	121/70085 (0.2%)	
Fasting blood glucose (mg/dL)				0.02
N (missing)	76801 (2749)	9362 (103)	67439 (2646)	
Median (25th, 75th)	121.0 (103.0, 155.0)	121.0 (104.0, 155.0)	121.0 (103.0, 155.0)	
Systolic blood pressure (mm Hg)				0.01
N (missing)	76202 (3348)	9303 (162)	66899 (3186)	
Median (25th, 75th)	156.0 (138.0, 178.0)	156.0 (138.0, 178.0)	156.0 (138.0, 178.0)	
Diastolic blood pressure (mm Hg)				0.01
N (missing)	76380 (3170)	9324 (141)	67056 (3029)	
Median (25th, 75th)	86.0 (76.0, 99.0)	86.0 (75.5, 99.0)	86.0 (76.0, 99.0)	
Heart rate (bpm)				-0.01
N (missing)	59603 (19947)	7088 (2377)	52515 (17570)	
Median (25th, 75th)	82.0 (71.0, 94.0)	82.0 (71.0, 94.0)	82.0 (71.0, 94.0)	
Creatinine (mg/dL)				0.01
N (missing)	54510 (25040)	6714 (2751)	47796 (22289)	
Median (25th, 75th)	1.0 (0.8, 1.2)	1.0 (0.8, 1.2)	1.0 (0.8, 1.2)	

© 2025 Rousseau JF et al. *JAMA Network Open*.

	All patients (N=79550)	Tenecteplase (N=9465)	Alteplase (N=70085)	Standardized difference
Time from LKW to arrival (minutes)				0.05
N (missing)	79550 (0)	9465 (0)	70085 (0)	
Median (25th, 75th)	68.0 (44.0, 113.0)	70.0 (46.0, 115.0)	68.0 (44.0, 112.0)	
Time from LKW to thrombolytic (minutes)				-0.07
N (missing)	79550 (0)	9465 (0)	70085 (0)	
Median (25th, 75th)	124.0 (90.0, 172.0)	120.0 (86.0, 169.0)	124.0 (90.0, 173.0)	
Brain imaging completed before thrombolytic	76998/79543 (96.8%)	9121/9464 (96.4%)	67877/70079 (96.9%)	-0.03
Brain imaging type				0.12
CT only	45692/76998 (59.3%)	5327/9121 (58.4%)	40365/67877 (59.5%)	
MRI only	336/76998 (0.4%)	103/9121 (1.1%)	233/67877 (0.3%)	
Both CT and MRI	26789/76998 (34.8%)	3054/9121 (33.5%)	23735/67877 (35.0%)	
Unknown	4181/76998 (5.4%)	637/9121 (7.0%)	3544/67877 (5.2%)	
Vascular or perfusion imaging before thrombolytic	50694/79529 (63.7%)	6340/9464 (67.0%)	44354/70065 (63.3%)	0.08
CTA	48573/50694 (95.8%)	5930/6340 (93.5%)	42643/44354 (96.1%)	-0.12
CT perfusion	20778/50694 (41.0%)	2435/6340 (38.4%)	18343/44354 (41.4%)	-0.06
MRA	1133/50694 (2.2%)	187/6340 (2.9%)	946/44354 (2.1%)	0.05
MR perfusion	167/50694 (0.3%)	97/6340 (1.5%)	70/44354 (0.2%)	0.15
DSA (catheter angiography)	1823/50694 (3.6%)	324/6340 (5.1%)	1499/44354 (3.4%)	0.09
Not documented	721/50694 (1.4%)	173/6340 (2.7%)	548/44354 (1.2%)	0.11
Stroke etiology				0.10
Large-artery atherosclerosis	10400/60221 (17.3%)	1181/7851 (15.0%)	9219/52370 (17.6%)	
Cardioembolism	15390/60221 (25.6%)	2246/7851 (28.6%)	13144/52370 (25.1%)	
Small-vessel occlusion	9199/60221 (15.3%)	1233/7851 (15.7%)	7966/52370 (15.2%)	
Stroke of other determined etiology	2370/60221 (3.9%)	346/7851 (4.4%)	2024/52370 (3.9%)	

© 2025 Rousseau JF et al. *JAMA Network Open*.

	All patients (N=79550)	Tenecteplase (N=9465)	Alteplase (N=70085)	Standardized difference
Cryptogenic Stroke	22862/60221 (38.0%)	2845/7851 (36.2%)	20017/52370 (38.2%)	
Large vessel occlusion	20682/79550 (26.0%)	2837/9465 (30.0%)	17845/70085 (25.5%)	0.10
LVO location among those with an LVO				0.01
Anterior	18905/20682 (91.4%)	2597/2837 (91.5%)	16308/17845 (91.4%)	
Posterior	1777/20682 (8.6%)	240/2837 (8.5%)	1537/17845 (8.6%)	
Potentially eligible for EVT	17036/79550 (21.4%)	2368/9465 (25.0%)	14668/70085 (20.9%)	0.10
Potentially eligible for and EVT performed	11315/17036 (66.4%)	1674/2368 (70.7%)	9641/14668 (65.7%)	0.11
EVT performed (among all patients)	11315/79550 (14.2%)	1674/9465 (17.7%)	9641/70085 (13.8%)	0.11
Patient location when stroke symptoms discovered				0.03
Not in a healthcare setting	76084/79550 (95.6%)	9089/9465 (96.0%)	66995/70085 (95.6%)	
Chronic healthcare facility	2591/79550 (3.3%)	273/9465 (2.9%)	2318/70085 (3.3%)	
Outpatient healthcare setting	858/79550 (1.1%)	100/9465 (1.1%)	758/70085 (1.1%)	
Not documented or cannot be determined	17/79550 (0.0%)	3/9465 (0.0%)	14/70085 (0.0%)	
**COVID-19 positive on admission**	2286/79550 (2.9%)	325/9465 (3.4%)	1961/70085 (2.8%)	0.04

© 2025 Rousseau JF et al. *JAMA Network Open*.

eTable 2. Demographic table for EVT Cohort (Potentially eligible for and EVT performed) is presented below

	EVT Cohort (N=11315)	Tenecteplase (N=1674)	Alteplase (N=9641)	Standardized difference
**Age (years)**				0.04
N (missing)	11315 (0)	1674 (0)	9641 (0)	
Mean (SD)	69.6 (14.9)	70.2 (14.7)	69.5 (14.9)	
Median (25th, 75th)	71.0 (60.0, 81.0)	72.0 (61.0, 81.0)	71.0 (60.0, 81.0)	
Range	(18.0, 110.0)	(20.0, 110.0)	(18.0, 105.0)	
**Sex**				-0.02
Female	5594/11315 (49.4%)	812/1674 (48.5%)	4782/9641 (49.6%)	
Male	5721/11315 (50.6%)	862/1674 (51.5%)	4859/9641 (50.4%)	
**Race/ethnicity**				0.12
White	7082/11315 (62.6%)	1107/1674 (66.1%)	5975/9641 (62.0%)	
Black	1875/11315 (16.6%)	224/1674 (13.4%)	1651/9641 (17.1%)	
Hispanic (any race)	1069/11315 (9.4%)	146/1674 (8.7%)	923/9641 (9.6%)	
Asian	484/11315 (4.3%)	79/1674 (4.7%)	405/9641 (4.2%)	
Other (includes unable to be determined)	805/11315 (7.1%)	118/1674 (7.0%)	687/9641 (7.1%)	
**Body mass index (kg/m^2)**				0.03
N (missing)	9580 (1735)	1459 (215)	8121 (1520)	
Mean (SD)	28.9 (6.9)	29.0 (6.8)	28.9 (6.9)	
Median (25th, 75th)	27.8 (24.3, 32.2)	28.1 (24.2, 32.6)	27.8 (24.3, 32.2)	
Range	(12.1, 84.7)	(12.5, 69.0)	(12.1, 84.7)	
**Homeless**	13/11315 (0.1%)	1/1674 (0.1%)	12/9641 (0.1%)	-0.02
**Insurance type**				0.11
Private/VA/Champus/Other insurance	3432/11315 (30.3%)	458/1674 (27.4%)	2974/9641 (30.8%)	
Medicaid	1509/11315 (13.3%)	240/1674 (14.3%)	1269/9641 (13.2%)	
Medicare	4220/11315 (37.3%)	688/1674 (41.1%)	3532/9641 (36.6%)	
Self-pay/No insurance	517/11315 (4.6%)	66/1674 (3.9%)	451/9641 (4.7%)	
Other/Not documented/Unable to determine	1637/11315 (14.5%)	222/1674 (13.3%)	1415/9641 (14.7%)	

© 2025 Rousseau JF et al. *JAMA Network Open*.

	EVT Cohort (N=11315)	Tenecteplase (N=1674)	Alteplase (N=9641)	Standardized difference
**Past medical history**				
Ambulatory status before current event				0.07
Able to ambulate independently (no help from another person) w/ or w/o device	8053/11315 (71.2%)	1232/1674 (73.6%)	6821/9641 (70.7%)	
With assistance (from person)	337/11315 (3.0%)	53/1674 (3.2%)	284/9641 (2.9%)	
Unable to ambulate	103/11315 (0.9%)	16/1674 (1.0%)	87/9641 (0.9%)	
Not documented	2822/11315 (24.9%)	373/1674 (22.3%)	2449/9641 (25.4%)	
Alcohol/drug use disorder	979/11315 (8.7%)	138/1674 (8.2%)	841/9641 (8.7%)	-0.02
Atrial fib/flutter	2481/11315 (21.9%)	381/1674 (22.8%)	2100/9641 (21.8%)	0.02
Carotid stenosis	286/11315 (2.5%)	48/1674 (2.9%)	238/9641 (2.5%)	0.03
CAD/prior MI	2225/11315 (19.7%)	327/1674 (19.5%)	1898/9641 (19.7%)	-0.00
Diabetes mellitus	2805/11315 (24.8%)	409/1674 (24.4%)	2396/9641 (24.9%)	-0.01
Dyslipidemia	5169/11315 (45.7%)	801/1674 (47.8%)	4368/9641 (45.3%)	0.05
Heart failure	1290/11315 (11.4%)	190/1674 (11.4%)	1100/9641 (11.4%)	-0.00
Hypertension	7994/11315 (70.6%)	1177/1674 (70.3%)	6817/9641 (70.7%)	-0.01
Peripheral vascular disease	365/11315 (3.2%)	63/1674 (3.8%)	302/9641 (3.1%)	0.04
Prior ischemic stroke	1170/11315 (10.3%)	183/1674 (10.9%)	987/9641 (10.2%)	0.02
Renal insufficiency	875/11315 (7.7%)	130/1674 (7.8%)	745/9641 (7.7%)	0.00
Sleep apnea	565/11315 (5.0%)	94/1674 (5.6%)	471/9641 (4.9%)	0.03
Smoker	1884/11315 (16.7%)	292/1674 (17.4%)	1592/9641 (16.5%)	0.03
**Medications before admission**				
Antiplatelet	3786/11315 (33.5%)	589/1674 (35.2%)	3197/9641 (33.2%)	0.04
Anticoagulant	616/11315 (5.4%)	105/1674 (6.3%)	511/9641 (5.3%)	0.04
Antihypertensive	5813/10001 (58.1%)	847/1438 (58.9%)	4966/8563 (58.0%)	0.02
**Arrival information**				
NIHSS				0.07

© 2025 Rousseau JF et al. *JAMA Network Open*.

	EVT Cohort (N=11315)	Tenecteplase (N=1674)	Alteplase (N=9641)	Standardized difference
N (missing)	11315 (0)	1674 (0)	9641 (0)	
Mean (SD)	17.3 (6.6)	17.7 (6.6)	17.2 (6.6)	
Median (25th, 75th)	17.0 (12.0, 22.0)	17.0 (13.0, 22.0)	17.0 (12.0, 22.0)	
Range	(6.0, 42.0)	(6.0, 40.0)	(6.0, 42.0)	
Off-hour arrival (Regular Hour: 7AM-6PM, M-F, non-holiday)	6273/11315 (55.4%)	923/1674 (55.1%)	5350/9641 (55.5%)	-0.01
Patient arrival				0.08
EMS from home/scene	10783/11315 (95.3%)	1608/1674 (96.1%)	9175/9641 (95.2%)	
Private transport/taxi/other from home/scene	461/11315 (4.1%)	57/1674 (3.4%)	404/9641 (4.2%)	
Not documented or unknown	51/11315 (0.5%)	3/1674 (0.2%)	48/9641 (0.5%)	
Mobile Stroke Unit	20/11315 (0.2%)	6/1674 (0.4%)	14/9641 (0.1%)	
Fasting blood glucose (mg/dL)				-0.01
N (missing)	11037 (278)	1658 (16)	9379 (262)	
Median (25th, 75th)	123.0 (105.0, 153.0)	122.0 (106.0, 152.0)	123.0 (105.0, 153.0)	
Systolic blood pressure (mm Hg)				-0.01
N (missing)	11022 (293)	1647 (27)	9375 (266)	
Median (25th, 75th)	150.0 (133.0, 170.0)	150.0 (133.0, 170.0)	150.0 (133.0, 171.0)	
Diastolic blood pressure (mm Hg)				0.01
N (missing)	11025 (290)	1644 (30)	9381 (260)	
Median (25th, 75th)	84.0 (74.0, 97.0)	84.0 (74.0, 98.0)	84.0 (74.0, 97.0)	
Heart rate (bpm)				-0.05
N (missing)	8625 (2690)	1341 (333)	7284 (2357)	
Median (25th, 75th)	81.0 (70.0, 95.0)	80.0 (70.0, 94.0)	81.0 (70.0, 95.0)	
Creatinine (mg/dL)				-0.01
N (missing)	8036 (3279)	1279 (395)	6757 (2884)	

© 2025 Rousseau JF et al. *JAMA Network Open*.

	EVT Cohort (N=11315)	Tenecteplase (N=1674)	Alteplase (N=9641)	Standardized difference
Median (25th, 75th)	1.0 (0.8, 1.2)	1.0 (0.8, 1.2)	1.0 (0.8, 1.2)	
Time from LKW to arrival (minutes)				0.08
N (missing)	11315 (0)	1674 (0)	9641 (0)	
Median (25th, 75th)	62.0 (41.0, 102.0)	66.0 (44.0, 105.0)	61.0 (41.0, 102.0)	
Time from LKW to thrombolytic (minutes)				0.01
N (missing)	11315 (0)	1674 (0)	9641 (0)	
Median (25th, 75th)	104.0 (77.0, 148.0)	105.0 (77.0, 148.0)	104.0 (77.0, 148.0)	
Brain imaging completed before thrombolytic	10885/11315 (96.2%)	1614/1674 (96.4%)	9271/9641 (96.2%)	0.01
Brain imaging type				0.13
CT only	6826/10885 (62.7%)	1005/1614 (62.3%)	5821/9271 (62.8%)	
MRI only	53/10885 (0.5%)	21/1614 (1.3%)	32/9271 (0.3%)	
Both CT and MRI	3391/10885 (31.2%)	476/1614 (29.5%)	2915/9271 (31.4%)	
Unknown	615/10885 (5.6%)	112/1614 (6.9%)	503/9271 (5.4%)	
Vascular or perfusion imaging before thrombolytic	8839/11311 (78.1%)	1342/1674 (80.2%)	7497/9637 (77.8%)	0.06
CTA	8416/8839 (95.2%)	1245/1342 (92.8%)	7171/7497 (95.7%)	-0.12
CT perfusion	4465/8839 (50.5%)	636/1342 (47.4%)	3829/7497 (51.1%)	-0.07
MRA	170/8839 (1.9%)	35/1342 (2.6%)	135/7497 (1.8%)	0.06
MR perfusion	26/8839 (0.3%)	16/1342 (1.2%)	10/7497 (0.1%)	0.13
DSA (catheter angiography)	1371/8839 (15.5%)	258/1342 (19.2%)	1113/7497 (14.8%)	0.12
Not documented	128/8839 (1.4%)	50/1342 (3.7%)	78/7497 (1.0%)	0.18
Stroke etiology				0.16
Large-artery atherosclerosis	2641/10098 (26.2%)	326/1570 (20.8%)	2315/8528 (27.1%)	
Cardioembolism	4203/10098 (41.6%)	704/1570 (44.8%)	3499/8528 (41.0%)	
Small-vessel occlusion	127/10098 (1.3%)	25/1570 (1.6%)	102/8528 (1.2%)	

© 2025 Rousseau JF et al. *JAMA Network Open*.

	EVT Cohort (N=11315)	Tenecteplase (N=1674)	Alteplase (N=9641)	Standardized difference
Stroke of other determined etiology	458/10098 (4.5%)	66/1570 (4.2%)	392/8528 (4.6%)	
Cryptogenic Stroke	2669/10098 (26.4%)	449/1570 (28.6%)	2220/8528 (26.0%)	
Large vessel occlusion	11315/11315 (100.0%)	1674/1674 (100.0%)	9641/9641 (100.0%)	0.00
LVO location among those with an LVO				-0.00
Anterior	10716/11315 (94.7%)	1584/1674 (94.6%)	9132/9641 (94.7%)	
Posterior	599/11315 (5.3%)	90/1674 (5.4%)	509/9641 (5.3%)	
Potentially eligible for EVT	11315/11315 (100.0%)	1674/1674 (100.0%)	9641/9641 (100.0%)	0.00
Potentially eligible for and EVT performed	11315/11315 (100.0%)	1674/1674 (100.0%)	9641/9641 (100.0%)	0.00
Patient location when stroke symptoms discovered				0.03
Not in a healthcare setting	10964/11315 (96.9%)	1624/1674 (97.0%)	9340/9641 (96.9%)	
Chronic healthcare facility	271/11315 (2.4%)	36/1674 (2.2%)	235/9641 (2.4%)	
Outpatient healthcare setting	79/11315 (0.7%)	14/1674 (0.8%)	65/9641 (0.7%)	
Not documented or cannot be determined	1/11315 (0.0%)	0/1674 (0.0%)	1/9641 (0.0%)	
**COVID-19 positive on admission**	496/11315 (4.4%)	83/1674 (5.0%)	413/9641 (4.3%)	0.03

© 2025 Rousseau JF et al. *JAMA Network Open*.

eTable 3. Demographic table for LVO non-EVT Cohort (Potentially eligible for EVT and EVT not performed) is presented below

	**LVO non-EVT Cohort (N=5721)**	**Tenecteplase (N=694)**	**Alteplase (N=5027)**	**Standardized difference**
**Age (years)**				0.11
N (missing)	5721 (0)	694 (0)	5027 (0)	
Mean (SD)	75.1 (14.8)	76.3 (15.1)	74.9 (14.8)	
Median (25th, 75th)	77.0 (65.0, 87.0)	79.0 (66.0, 89.0)	77.0 (65.0, 87.0)	
Range	(19.0, 107.0)	(25.0, 102.0)	(19.0, 107.0)	
**Sex**				0.08
Female	3041/5721 (53.2%)	393/694 (56.6%)	2648/5027 (52.7%)	
Male	2680/5721 (46.8%)	301/694 (43.4%)	2379/5027 (47.3%)	
**Race/ethnicity**				0.17
White	3753/5721 (65.6%)	494/694 (71.2%)	3259/5027 (64.8%)	
Black	900/5721 (15.7%)	85/694 (12.2%)	815/5027 (16.2%)	
Hispanic (any race)	517/5721 (9.0%)	47/694 (6.8%)	470/5027 (9.3%)	
Asian	235/5721 (4.1%)	33/694 (4.8%)	202/5027 (4.0%)	
Other (includes unable to be determined)	316/5721 (5.5%)	35/694 (5.0%)	281/5027 (5.6%)	
**Body mass index (kg/m^2)**				-0.020
N (missing)	4964 (757)	612 (82)	4352 (675)	
Mean (SD)	27.9 (6.8)	27.5 (6.3)	27.9 (6.8)	
Median (25th, 75th)	26.9 (23.3, 31.2)	27.0 (23.4, 31.0)	26.9 (23.2, 31.3)	
Range	(12.4, 85.3)	(12.4, 58.0)	(12.8, 85.3)	
**Homeless**	9/5721 (0.2%)	1/694 (0.1%)	8/5027 (0.2%)	-0.004
**Insurance type**				0.097
Private/VA/Champus/Other insurance	1616/5721 (28.2%)	187/694 (26.9%)	1429/5027 (28.4%)	
Medicaid	708/5721 (12.4%)	74/694 (10.7%)	634/5027 (12.6%)	
Medicare	2405/5721 (42.0%)	296/694 (42.7%)	2109/5027 (42.0%)	
Self-pay/No insurance	170/5721 (3.0%)	20/694 (2.9%)	150/5027 (3.0%)	
Other/Not documented/Unable to determine	822/5721 (14.4%)	117/694 (16.9%)	705/5027 (14.0%)	

© 2025 Rousseau JF et al. *JAMA Network Open*.

	LVO non-EVT Cohort (N=5721)	Tenecteplase (N=694)	Alteplase (N=5027)	Standardized difference
**Past medical history**				
Ambulatory status before current event				0.11
Able to ambulate independently (no help from another person) w/ or w/o device	3716/5721 (65.0%)	453/694 (65.3%)	3263/5027 (64.9%)	
With assistance (from person)	454/5721 (7.9%)	71/694 (10.2%)	383/5027 (7.6%)	
Unable to ambulate	214/5721 (3.7%)	28/694 (4.0%)	186/5027 (3.7%)	
Not documented	1337/5721 (23.4%)	142/694 (20.5%)	1195/5027 (23.8%)	
Alcohol/drug use disorder	426/5721 (7.4%)	54/694 (7.8%)	372/5027 (7.4%)	0.01
Atrial fib/flutter	1447/5721 (25.3%)	211/694 (30.4%)	1236/5027 (24.6%)	0.13
Carotid stenosis	230/5721 (4.0%)	31/694 (4.5%)	199/5027 (4.0%)	0.03
CAD/prior MI	1344/5721 (23.5%)	159/694 (22.9%)	1185/5027 (23.6%)	-0.02
Diabetes mellitus	1563/5721 (27.3%)	186/694 (26.8%)	1377/5027 (27.4%)	-0.01
Dyslipidemia	2863/5721 (50.0%)	379/694 (54.6%)	2484/5027 (49.4%)	0.10
Heart failure	785/5721 (13.7%)	97/694 (14.0%)	688/5027 (13.7%)	0.01
Hypertension	4353/5721 (76.1%)	525/694 (75.6%)	3828/5027 (76.1%)	-0.01
Peripheral vascular disease	224/5721 (3.9%)	23/694 (3.3%)	201/5027 (4.0%)	-0.04
Prior ischemic stroke	849/5721 (14.8%)	105/694 (15.1%)	744/5027 (14.8%)	0.01
Renal insufficiency	643/5721 (11.2%)	78/694 (11.2%)	565/5027 (11.2%)	0.00
Sleep apnea	272/5721 (4.8%)	35/694 (5.0%)	237/5027 (4.7%)	0.02
Smoker	834/5721 (14.6%)	110/694 (15.9%)	724/5027 (14.4%)	0.04
**Medications before admission**				
Antiplatelet	2323/5721 (40.6%)	283/694 (40.8%)	2040/5027 (40.6%)	0.00
Anticoagulant	311/5721 (5.4%)	37/694 (5.3%)	274/5027 (5.5%)	-0.01
Antihypertensive	3316/5089 (65.2%)	387/595 (65.0%)	2929/4494 (65.2%)	-0.00
**Arrival information**				
NIHSS				0.02

© 2025 Rousseau JF et al. *JAMA Network Open*.

	LVO non-EVT Cohort (N=5721)	Tenecteplase (N=694)	Alteplase (N=5027)	Standardized difference
N (missing)	5721 (0)	694 (0)	5027 (0)	
Mean (SD)	15.3 (7.3)	15.4 (7.2)	15.3 (7.3)	
Median (25th, 75th)	14.0 (9.0, 20.0)	14.0 (9.0, 21.0)	14.0 (9.0, 20.0)	
Range	(6.0, 42.0)	(6.0, 39.0)	(6.0, 42.0)	
Off-hour arrival (Regular Hour: 7AM-6PM, M-F, non-holiday)	3331/5721 (58.2%)	430/694 (62.0%)	2901/5027 (57.7%)	0.09
Patient arrival				0.14
EMS from home/scene	5249/5721 (91.7%)	651/694 (93.8%)	4598/5027 (91.5%)	
Private transport/taxi/other from home/scene	429/5721 (7.5%)	42/694 (6.1%)	387/5027 (7.7%)	
Not documented or unknown	35/5721 (0.6%)	0/694 (0.0%)	35/5027 (0.7%)	
Mobile Stroke Unit	8/5721 (0.1%)	1/694 (0.1%)	7/5027 (0.1%)	
Fasting blood glucose (mg/dL)				-0.04
N (missing)	5566 (155)	690 (4)	4876 (151)	
Median (25th, 75th)	125.0 (106.0, 157.0)	124.0 (105.0, 155.0)	125.0 (106.0, 157.0)	
Systolic blood pressure (mm Hg)				-0.02
N (missing)	5544 (177)	684 (10)	4860 (167)	
Median (25th, 75th)	154.0 (136.0, 175.0)	154.0 (137.0, 173.0)	154.0 (136.0, 176.0)	
Diastolic blood pressure (mm Hg)				-0.054
N (missing)	5557 (164)	685 (9)	4872 (155)	
Median (25th, 75th)	85.0 (73.0, 98.0)	83.0 (72.0, 98.0)	85.0 (74.0, 98.0)	
Heart rate (bpm)				0.03
N (missing)	4546 (1175)	542 (152)	4004 (1023)	
Median (25th, 75th)	82.0 (71.0, 95.0)	82.0 (71.0, 95.0)	82.0 (71.0, 95.0)	
Creatinine (mg/dL)				-0.05
N (missing)	4226 (1495)	535 (159)	3691 (1336)	

© 2025 Rousseau JF et al. *JAMA Network Open*.

	LVO non-EVT Cohort (N=5721)	Tenecteplase (N=694)	Alteplase (N=5027)	Standardized difference
Median (25th, 75th)	1.0 (0.8, 1.3)	1.0 (0.8, 1.3)	1.0 (0.8, 1.3)	
Time from LKW to arrival (minutes)				0.04
N (missing)	5721 (0)	694 (0)	5027 (0)	
Median (25th, 75th)	67.0 (44.0, 110.0)	66.0 (46.0, 110.0)	67.0 (43.0, 110.0)	
Time from LKW to thrombolytic (minutes)				-0.04
N (missing)	5721 (0)	694 (0)	5027 (0)	
Median (25th, 75th)	120.0 (87.0, 166.0)	119.0 (85.0, 161.0)	120.0 (87.0, 167.0)	
Brain imaging completed before thrombolytic	5530/5721 (96.7%)	667/694 (96.1%)	4863/5027 (96.7%)	-0.03
Brain imaging type				0.12
CT only	3376/5530 (61.0%)	415/667 (62.2%)	2961/4863 (60.9%)	
MRI only	33/5530 (0.6%)	10/667 (1.5%)	23/4863 (0.5%)	
Both CT and MRI	1855/5530 (33.5%)	207/667 (31.0%)	1648/4863 (33.9%)	
Unknown	266/5530 (4.8%)	35/667 (5.2%)	231/4863 (4.8%)	
Vascular or perfusion imaging before thrombolytic	4342/5719 (75.9%)	542/694 (78.1%)	3800/5025 (75.6%)	0.06
CTA	4173/4342 (96.1%)	502/542 (92.6%)	3671/3800 (96.6%)	-0.18
CT perfusion	2095/4342 (48.2%)	263/542 (48.5%)	1832/3800 (48.2%)	0.01
MRA	108/4342 (2.5%)	20/542 (3.7%)	88/3800 (2.3%)	0.08
MR perfusion	19/4342 (0.4%)	14/542 (2.6%)	5/3800 (0.1%)	0.21
DSA (catheter angiography)	120/4342 (2.8%)	19/542 (3.5%)	101/3800 (2.7%)	0.05
Not documented	47/4342 (1.1%)	11/542 (2.0%)	36/3800 (0.9%)	0.09
Stroke etiology				0.19
Large-artery atherosclerosis	1478/4893 (30.2%)	150/633 (23.7%)	1328/4260 (31.2%)	
Cardioembolism	1784/4893 (36.5%)	263/633 (41.5%)	1521/4260 (35.7%)	
Small-vessel occlusion	162/4893 (3.3%)	29/633 (4.6%)	133/4260 (3.1%)	

© 2025 Rousseau JF et al. *JAMA Network Open*.

	LVO non-EVT Cohort (N=5721)	Tenecteplase (N=694)	Alteplase (N=5027)	Standardized difference
Stroke of other determined etiology	153/4893 (3.1%)	24/633 (3.8%)	129/4260 (3.0%)	
Cryptogenic Stroke	1316/4893 (26.9%)	167/633 (26.4%)	1149/4260 (27.0%)	
Large vessel occlusion	5721/5721 (100.0%)	694/694 (100.0%)	5027/5027 (100.0%)	0.00
LVO location among those with an LVO				-0.01
Anterior	5215/5721 (91.2%)	631/694 (90.9%)	4584/5027 (91.2%)	
Posterior	506/5721 (8.8%)	63/694 (9.1%)	443/5027 (8.8%)	
Potentially eligible for EVT	5721/5721 (100.0%)	694/694 (100.0%)	5027/5027 (100.0%)	0.00
Potentially eligible for and EVT performed	0/5721 (0.0%)	0/694 (0.0%)	0/5027 (0.0%)	0.00
Patient location when stroke symptoms discovered				0.04
Not in a healthcare setting	5280/5721 (92.3%)	646/694 (93.1%)	4634/5027 (92.2%)	
Chronic healthcare facility	403/5721 (7.0%)	45/694 (6.5%)	358/5027 (7.1%)	
Outpatient healthcare setting	38/5721 (0.7%)	3/694 (0.4%)	35/5027 (0.7%)	
**COVID-19 positive on admission**	218/5721 (3.8%)	27/694 (3.9%)	191/5027 (3.8%)	0.01

© 2025 Rousseau JF et al. *JAMA Network Open*.

**eTable 4.** Comparison of patient characteristics between those with discharge mRS available vs. missing			
	**Discharge mRS available (N=55492)**	**Missing discharge mRS (N=24058)**	**Standardized difference**
**Tenecteplase administered**	7278/55492 (13.1%)	2187/24058 (9.1%)	0.13
**Age at admission (years)**			0.03
N (missing)	55492 (0)	24058 (0)	
Mean (SD)	68.8 (14.8)	68.3 (14.9)	
Median (25th, 75th)	70.0 (59.0, 80.0)	69.0 (58.0, 79.0)	
Range	(18.0, 110.0)	(18.0, 105.0)	
**Sex**			-0.02
Female	26751/55492 (48.2%)	11845/24058 (49.2%)	
Male	28741/55492 (51.8%)	12213/24058 (50.8%)	
**Race/ethnicity**			0.10
White	36254/55492 (65.3%)	16831/24058 (70.0%)	
Black	9254/55492 (16.7%)	3367/24058 (14.0%)	
Hispanic (any race)	5343/55492 (9.6%)	1992/24058 (8.3%)	
Asian	1930/55492 (3.5%)	708/24058 (2.9%)	
Other (includes unable to be determined)	2711/55492 (4.9%)	1160/24058 (4.8%)	
**Body mass index (kg/m^2)**			-0.03
N (missing)	48030 (7462)	19961 (4097)	
Mean (SD)	29.2 (7.0)	29.5 (7.2)	
Median (25th, 75th)	28.2 (24.5, 32.8)	28.4 (24.6, 33.2)	
Range	(10.4, 89.1)	(10.0, 97.8)	
**Homeless**	67/55492 (0.1%)	10/24058 (0.0%)	0.03
**Insurance type**			0.25
Private/VA/Champus/Other insurance	18219/55492 (32.8%)	6904/24058 (28.7%)	
Medicaid	6821/55492 (12.3%)	2678/24058 (11.1%)	
Medicare	20704/55492 (37.3%)	7965/24058 (33.1%)	
Self-pay/No insurance	2209/55492 (4.0%)	1011/24058 (4.2%)	
Other/Not documented/Unable to determine	7539/55492 (13.6%)	5500/24058 (22.9%)	
**Past medical history**			
Ambulatory status before the current event			0.22

© 2025 Rousseau JF et al. *JAMA Network Open*.

**eTable 4.** Comparison of patient characteristics between those with discharge mRS available vs. missing			
Able to ambulate independently (no help from another person) w/ or w/o device	39426/55492 (71.0%)	14977/24058 (62.3%)	
With assistance (from person)	2192/55492 (4.0%)	726/24058 (3.0%)	
Unable to ambulate	735/55492 (1.3%)	369/24058 (1.5%)	
Not documented	13139/55492 (23.7%)	7986/24058 (33.2%)	
Alcohol/drug use disorder	4468/55492 (8.1%)	1817/24058 (7.6%)	0.02
Atrial fib/flutter	7548/55492 (13.6%)	3081/24058 (12.8%)	0.02
Carotid stenosis	1538/55492 (2.8%)	674/24058 (2.8%)	-0.00
CAD/prior MI	11129/55492 (20.1%)	4850/24058 (20.2%)	-0.00
Diabetes mellitus	16552/55492 (29.8%)	7259/24058 (30.2%)	-0.01
Dyslipidemia	27534/55492 (49.6%)	11753/24058 (48.9%)	0.02
Heart failure	5058/55492 (9.1%)	2113/24058 (8.8%)	0.01
Hypertension	40499/55492 (73.0%)	17415/24058 (72.4%)	0.01
Peripheral vascular disease	1676/55492 (3.0%)	720/24058 (3.0%)	0.00
Prior ischemic stroke	7837/55492 (14.1%)	3189/24058 (13.3%)	0.03
Renal insufficiency	4784/55492 (8.6%)	2228/24058 (9.3%)	-0.02
Sleep apnea	3669/55492 (6.6%)	1789/24058 (7.4%)	-0.03
Smoker	9241/55492 (16.7%)	4312/24058 (17.9%)	-0.03
**Medications before admission**			
Antiplatelet	21834/55492 (39.3%)	9153/24058 (38.0%)	0.03
Anticoagulant	1881/55492 (3.4%)	745/24058 (3.1%)	0.02
Antihypertensive	29904/49380 (60.6%)	11469/19218 (59.7%)	0.02
**Arrival information**			
NIHSS			0.10
N (missing)	55377 (115)	23997 (61)	
Mean (SD)	9.3 (7.5)	8.5 (7.0)	
Median (25th, 75th)	7.0 (4.0, 14.0)	6.0 (3.0, 12.0)	
Range	(0.0, 42.0)	(0.0, 42.0)	
Off-hour arrival (Regular Hour: 7AM-6PM, M-F, non-holiday)	30009/55492 (54.1%)	12867/24058 (53.5%)	0.01
Patient arrival			0.14
EMS from home/scene	43272/55492 (78.0%)	17336/24058 (72.1%)	
Private transport/taxi/other from home/scene	11823/55492 (21.3%)	6545/24058 (27.2%)	

© 2025 Rousseau JF et al. *JAMA Network Open*.

**eTable 4.** Comparison of patient characteristics between those with discharge mRS available vs. missing			
Not documented or unknown	280/55492 (0.5%)	144/24058 (0.6%)	
Mobile Stroke Unit	117/55492 (0.2%)	33/24058 (0.1%)	
Fasting blood glucose (mg/dL)			0.03
N (missing)	54342 (1150)	22459 (1599)	
Mean (SD)	142.9 (69.2)	141.7 (69.0)	
Median (25th, 75th)	121.0 (103.0, 155.0)	120.0 (102.0, 153.0)	
Range	(40.0, 797.0)	(40.0, 779.0)	
Systolic blood pressure (mm Hg)			-0.01
N (missing)	54281 (1211)	21921 (2137)	
Mean (SD)	158.4 (29.5)	158.6 (29.5)	
Median (25th, 75th)	156.0 (138.0, 178.0)	156.0 (138.0, 178.0)	
Range	(51.0, 250.0)	(50.0, 250.0)	
Diastolic blood pressure (mm Hg)			-0.01
N (missing)	54407 (1085)	21973 (2085)	
Mean (SD)	87.8 (18.7)	88.1 (19.0)	
Median (25th, 75th)	86.0 (76.0, 99.0)	86.0 (76.0, 99.0)	
Range	(20.0, 200.0)	(20.0, 200.0)	
Heart rate (bpm)			-0.00
N (missing)	43175 (12317)	16428 (7630)	
Mean (SD)	83.8 (18.1)	83.8 (17.8)	
Median (25th, 75th)	82.0 (71.0, 94.0)	82.0 (71.0, 94.0)	
Range	(30.0, 198.0)	(31.0, 197.0)	
Creatinine (mg/dL)			0.02
N (missing)	40337 (15155)	14173 (9885)	
Mean (SD)	1.2 (1.0)	1.2 (0.9)	
Median (25th, 75th)	1.0 (0.8, 1.2)	1.0 (0.8, 1.2)	
Range	(0.1, 20.0)	(0.1, 20.0)	
Time from LKW to arrival (minutes)			0.05
N (missing)	55492 (0)	24058 (0)	
Mean (SD)	83.8 (59.2)	81.4 (53.7)	
Median (25th, 75th)	69.0 (44.0, 113.0)	67.0 (43.0, 111.0)	
Range	(0.0, 2999.0)	(0.0, 2190.0)	

© 2025 Rousseau JF et al. *JAMA Network Open*.

**eTable 4.** Comparison of patient characteristics between those with discharge mRS available vs. missing			
Time from LKW to thrombolytic (minutes)			-0.04
N (missing)	55492 (0)	24058 (0)	
Mean (SD)	133.9 (56.5)	135.8 (56.2)	
Median (25th, 75th)	123.0 (89.0, 172.0)	125.0 (91.0, 175.0)	
Range	(9.0, 270.0)	(20.0, 270.0)	
Brain imaging completed before thrombolytic	53952/55488 (97.2%)	23046/24055 (95.8%)	0.08
Brain imaging type			0.09
CT only	32596/53952 (60.4%)	13096/23046 (56.8%)	
MRI only	265/53952 (0.5%)	71/23046 (0.3%)	
Both CT and MRI	18147/53952 (33.6%)	8642/23046 (37.5%)	
Unknown	2944/53952 (5.5%)	1237/23046 (5.4%)	
Vascular or perfusion imaging before thrombolytic	36860/55475 (66.4%)	13834/24054 (57.5%)	0.19
CTA	35230/36860 (95.6%)	13343/13834 (96.5%)	-0.05
CT perfusion	16059/36860 (43.6%)	4719/13834 (34.1%)	0.20
MRA	790/36860 (2.1%)	343/13834 (2.5%)	-0.02
MR perfusion	148/36860 (0.4%)	19/13834 (0.1%)	0.05
DSA (catheter angiography)	1466/36860 (4.0%)	357/13834 (2.6%)	0.08
Not documented	511/36860 (1.4%)	210/13834 (1.5%)	-0.01
**Patient characteristics**			
Stroke etiology			0.08
Large-artery atherosclerosis (e.g., carotid or basilar artery stenosis)	7479/44292 (16.9%)	2921/15929 (18.3%)	
Cardioembolism (e.g., atrial fibrillation/flutter, prosthetic heart valve, recent MI)	11695/44292 (26.4%)	3695/15929 (23.2%)	
Small-vessel occlusion (e.g., Subcortical or brain stem lacunar infarction <1.5 cm)	6670/44292 (15.1%)	2529/15929 (15.9%)	
Stroke of other determined etiology (e.g., dissection, vasculopathy, hypercoagulable or hematologic disorders.	1766/44292 (4.0%)	604/15929 (3.8%)	
Cryptogenic Stroke (Stroke of undetermined etiology)	16682/44292 (37.7%)	6180/15929 (38.8%)	
Large vessel occlusion	15891/55492 (28.6%)	4791/24058 (19.9%)	0.21
LVO location among those with an LVO			0.01
Anterior	14533/15891 (91.5%)	4372/4791 (91.3%)	
Posterior	1358/15891 (8.5%)	419/4791 (8.7%)	

© 2025 Rousseau JF et al. *JAMA Network Open*.

**eTable 4.** Comparison of patient characteristics between those with discharge mRS available vs. missing			
Eligible for EVT	13198/55492 (23.8%)	3838/24058 (16.0%)	0.20
Underwent EVT	9031/13198 (68.4%)	2284/3838 (59.5%)	0.19
Underwent EVT (among all patients)	1674/9465 (17.7%)	9641/70085 (13.8%)	0.20
Patient location when stroke symptoms discovered			0.03
Not in a healthcare setting	53153/55492 (95.8%)	22931/24058 (95.3%)	
Chronic healthcare facility	1725/55492 (3.1%)	866/24058 (3.6%)	
Outpatient healthcare setting	606/55492 (1.1%)	252/24058 (1.0%)	
Not documented or cannot be determined	8/55492 (0.0%)	9/24058 (0.0%)	
**COVID-19 positive on admission**	1608/55492 (2.9%)	678/24058 (2.8%)	0.01

© 2025 Rousseau JF et al. *JAMA Network Open*.

eTable 5. Effectiveness and Safety Outcomes with Tenecteplase and Alteplase Among Potentially EVT-Eligible Patients Treated with EVT (EVT Cohort)

**Outcome**	**Total**	**Tenecteplase**	**Alteplase**	**Unadjusted OR**	**Adjusted OR**
Effectiveness Outcomes*					
mRS 0-2	2605/9031 (28.8%)	422/1411 (29.9%)	2183/7620 (28.6%)	1.06 (0.91, 1.24)	1.09 (0.92, 1.29)
mRS 0-1	1832/9031 (20.3%)	305/1411 (21.6%)	1527/7620 (20.0%)	1.13 (0.96, 1.34)	1.19 (0.99, 1.42)
Discharge Home	4359/11315 (38.5%)	627/1674 (37.5%)	3732/9641 (38.7%)	0.96 (0.85, 1.08)	0.99 (0.87, 1.13)
Independent Ambulation	3376/7813 (43.2%)	525/1204 (43.6%)	2851/6609 (43.1%)	0.99 (0.86, 1.15)	1.02 (0.87, 1.20)
Both Discharge Home + Independent Ambulation	2650/7964 (33.3%)	404/1217 (33.2%)	2246/6747 (33.3%)	1.00 (0.86, 1.16)	1.04 (0.89, 1.23)
Safety Outcomes					
sICH	683/11315 (6.0%)	83/1674 (5.0%)	600/9641 (6.2%)	0.80 (0.61, 1.03)	0.78 (0.59, 1.02)
SSH	105/11315 (0.9%)	16/1674 (1.0%)	89/9641 (0.9%)	---[1]	---
sICH + SSH	782/11315 (6.9%)	99/1674 (5.9%)	683/9641 (7.1%)	0.82 (0.65, 1.05)	0.80 (0.63, 1.03)
In-Hospital Mortality	1267/11315 (11.2%)	199/1674 (11.9%)	1068/9641 (11.1%)	1.09 (0.92, 1.30)	1.07 (0.89, 1.29)
In-Hospital Mortality + Discharge to Hospice	2243/11315 (19.8%)	348/1674 (20.8%)	1895/9641 (19.7%)	1.07 (0.94, 1.23)	1.05 (0.90, 1.21)
Post-treatment TICI score 2b or 3	9245/10024 (92.2%)	1411/1526 (92.5%)	7834/8498 (92.2%)	1.04 (0.83, 1.30)	1.03 (0.82, 1.29)

*For effectiveness outcomes data was missing in 0% of patients for discharge destination, 3.0% for ambulatory status at discharge among those documented to ambulate independently pre-stroke; 33.7% overall for ambulatory status at discharge, and 30.2% for mRS at discharge. Supplemental Table S2 compares demographic and clinical characteristics of patients with mRS non-missing and missing.
sICH: Symptomatic Intracranial Hemorrhage; SSH: Serious Systemic Hemorrhage; mRS: Modified Rankin Scale
[1]Models for SSH were not fit due to low event counts.

© 2025 Rousseau JF et al. *JAMA Network Open*.

eTable 6. Effectiveness and Safety Outcomes with Tenecteplase and Alteplase Among Potentially EVT-Eligible Patients Not Treated with EVT (LVO non-EVT Cohort)

Outcome	Total	Tenecteplase	Alteplase	Unadjusted OR	Adjusted OR
Effectiveness Outcomes*					
mRS 0-2	997/4167 (23.9%)	138/530 (26.0%)	859/3637 (23.6%)	1.14 (0.91, 1.43)	1.17 (0.91, 1.51)
mRS 0-1	712/4167 (17.1%)	100/530 (18.9%)	612/3637 (16.8%)	1.15 (0.90, 1.47)	1.13 (0.86, 1.49)
Discharge Home	2025/5721 (35.4%)	262/694 (37.8%)	1763/5027 (35.1%)	1.13 (0.96, 1.35)	1.26 (1.03, 1.53)
Independent Ambulation	1440/3608 (39.9%)	174/434 (40.1%)	1266/3174 (39.9%)	1.03 (0.83, 1.29)	1.05 (0.82, 1.34)
Both Discharge Home + Independent Ambulation	1130/3685 (30.7%)	141/444 (31.8%)	989/3241 (30.5%)	1.08 (0.86, 1.35)	1.12 (0.87, 1.44)
Safety Outcomes					
sICH	298/5721 (5.2%)	34/694 (4.9%)	264/5027 (5.3%)	0.93 (0.63, 1.36)	0.96 (0.65, 1.41)
SSH	36/5721 (0.6%)	2/694 (0.3%)	34/5027 (0.7%)	---[1]	---
sICH + SSH	330/5721 (5.8%)	36/694 (5.2%)	294/5027 (5.8%)	0.88 (0.60, 1.27)	0.90 (0.62, 1.32)
In-Hospital Mortality	716/5721 (12.5%)	65/694 (9.4%)	651/5027 (13.0%)	0.70 (0.53, 0.93)	0.63 (0.47, 0.85)
In-Hospital Mortality + Discharge to Hospice	1688/5721 (29.5%)	196/694 (28.2%)	1492/5027 (29.7%)	0.93 (0.77, 1.11)	0.78 (0.62, 0.97)

*For effectiveness outcomes data was missing in 0% of patients for discharge destination, 3.0% for ambulatory status at discharge among those documented to ambulate independently pre-stroke; 33.7% overall for ambulatory status at discharge, and 30.2% for mRS at discharge. Supplemental Table S2 compares demographic and clinical characteristics of patients with mRS non-missing and missing.
sICH: Symptomatic Intracranial Hemorrhage; SSH: Serious Systemic Hemorrhage; mRS: Modified Rankin Scale
[1]Models for SSH were not fit due to low event counts.

© 2025 Rousseau JF et al. *JAMA Network Open*.
