# Supplementary material for: Short-Term Safety and Effectiveness for Tenecteplase and Alteplase in Acute Ischemic Stroke
Source: JAMA Netw Open. 2025 Mar 12;8(3):e250548. doi: 10.1001/jamanetworkopen.2025.0548 (PMC11904722; doi:10.1001/jamanetworkopen.2025.0548)
Supplement: Supplement 2. — Data Sharing Statement [file jamanetwopen-e250548-s002.pdf]

Data Sharing Statement

Rousseau. Short-Term Safety and Effectiveness for Tenecteplase and Alteplase in Acute Ischemic Stroke. *JAMA Netw Open*. Published March 12, 2025.
doi:10.1001/jamanetworkopen.2025.0548

Data
Data available: No

Additional Information
Explanation for why data not available: AHA data is collected for clinical care and quality improvement, rather than primarily for research, data sharing agreements require an application process for other researchers to access the data.
